# AI-driven diagnostic and prognostic models for metabolic dysfunction-associated steatotic liver disease: insights from clinical, imaging, and multi-omics studies—a scoping review

**DOI:** 10.3389/fmed.2026.1875846

**Published:** 2026-08-27

**Authors:** M. Sujana, G. N. Vivekananda

**Affiliations:** School of Computer Science Engineering and Information Systems, Vellore Institute of Technology, Vellore, Tamil Nadu, India

**Keywords:** artificial intelligence, deep learning, imaging biomarkers, liver disease diagnosis, machine learning, MASLD, multi-omics, NAFLD

## Abstract

Metabolic dysfunction-associated steatotic liver disease (MASLD), formerly known as non-alcoholic fatty liver disease (NAFLD), is the most common chronic liver disease around the world, affecting 33.6% of the adult population (95% CI: 28.1%–39.5%; *I*^2^ = 99.9%), or roughly one in three. The extent of the liver damage is variable, from simple steatosis to metabolic dysfunction-associated steatohepatitis (MASH, formerly NASH), cirrhosis and hepatocellular carcinoma (HCC). Early diagnosis is essential to prevent serious liver damage. Traditional diagnostic techniques such as liver biopsy, imaging, and biomarker testing are all invasive, costly, reduced sensitive to early-stage disease, and they also have variability among observers. Modern diagnostic and prognostic approaches based on the principles of Artificial Intelligence (AI) and specifically on machine learning (ML) and deep learning (DL) have enabled multimodal approaches integrating clinical, imaging and molecular data. This scoping review conducted per PRISMA-ScR guidelines, synthesizes findings from 73 studies (search window 2020–2026) across three dimensions: clinical data driven models, imaging-based classifiers (ultrasound, CT and MRI), and multi-omics (genomics, transcriptomics and proteomics) techniques. Moreover, emergence of models such as U-Net and LiverNet 2.x, classification models like DeepLiverNet and BiLSTM models, as well as transformer frameworks and the identification of biomarkers models are also described. This study also investigates challenges such as data heterogeneity, data interpretability, fairness and real-world clinical application. Finally, important areas of research opportunities and future directions are highlighted to present a developing clinically applicable, explainable and ethical AI solutions to manage MASLD.

## Highlights

- Provides a comprehensive review of AI-driven diagnostic and prognostic models for MASLD, integrating clinical, imaging, and multi-omics perspectives.
- Critically compares imaging-based deep learning models, clinical risk stratification algorithms, and omics-integrated pipelines, highlighting their relative strengths and limitations.
- Identifies major technical and clinical challenges including dataset heterogeneity, lack of interpretability, limited external validation, and deployment barriers in real-world settings.
- Discusses emerging solutions such as federated learning, explainable AI frameworks, and multi-modal data fusion for improving generalizability and clinical adoption.
- Proposes a forward-looking roadmap for developing clinically deployable, ethically sound, and regulation-ready AI systems in MASLD care.

## 1 Introduction

Metabolic dysfunction-associated steatotic liver disease [MASLD, formerly non-alcoholic fatty liver disease (NAFLD)], redefined under the international 2023 Delphi consensus nomenclature to reflect its strong association with metabolic dysfunction, is now recognized as the most common chronic liver disorder globally, affecting approximately 33.6% of the adult population (95% CI: 28.1%–39.5%) and approximately 70.2% of individuals with metabolic syndrome (95% CI: 66.1%–73.9%; *I*^2^ = 65.4%) (1, 2). Prevalence estimates under the current MASLD criteria are not directly comparable to legacy NAFLD prevalence estimates, given the shift from an exclusion-based to an inclusion-based case definition. As a progressive and multifaceted disease, MASLD encompasses a pathological continuum from simple steatosis to metabolic dysfunction-associated steatohepatitis (MASH), advancing through fibrosis, cirrhosis, and ultimately hepatocellular carcinoma (HCC). Despite this well-documented trajectory, MASLD remains underdiagnosed and undertreated due to the asymptomatic nature of its early stages and the limitations of existing diagnostic tools (3).

Standard diagnostic approaches like liver biopsy, although considered the gold standard, are invasive, costly, and impractical for routine surveillance or population-wide screening. Non-invasive alternatives such as ultrasonography and transient elastography are more scalable but have limited sensitivity, especially in detecting mild steatosis or early fibrosis. Imaging techniques such as computed tomography (CT) and magnetic resonance imaging (MRI) improve diagnostic accuracy but remain cost-prohibitive and less accessible in low-resource settings (4). Biomarker-based scoring systems like the NAFLD Fibrosis Score (NFS), Fibrosis-4 (FIB-4), and AST to Platelet Ratio Index (APRI) offer some clinical utility but are often confounded by comorbidities and lack personalized predictive capability (5).

In response to these diagnostic challenges, researchers have begun using Artificial Intelligence (AI) to analyze large-scale multimodal data including imaging, clinical biomarkers, and multi-omics for early detection and disease stratification. By integrating machine learning (ML) and deep learning (DL) algorithms, AI systems can uncover subtle patterns and non-linear relationships in data that traditional statistical models often overlook. Recently, convolutional neural networks (CNNs) and U-Net based studies have reported dice similarity coefficients and AUROC values approaching or exceeding 0.90 for the performance of liver segmentation and fatty liver detection in several published studies. This shows their usefulness for precise MASLD diagnosis (6, 7).

However, the current landscape of AI research in MASLD is fragmented, with studies varying in methodology, input modalities, evaluation metrics, and clinical contexts. Some efforts focus narrowly on radiomics, while others attempt to integrate omics without addressing interpretability, reproducibility, or clinical utility. Given the complexity of MASLD pathophysiology and the heterogeneity of data sources, a holistic and comparative synthesis is necessary to understand the maturity, limitations, and translational readiness of AI-driven solutions.

This review addresses that need by adopting a scoping review methodology, which is particularly well-suited for emerging interdisciplinary fields. Unlike systematic reviews that answer specific clinical questions or meta-analyses that aggregate effect sizes, a scoping review allows for mapping the breadth and depth of available research. It facilitates exploration of themes, model types, and data domains without imposing narrow inclusion restrictions (8).

### 1.1 Nomenclature transition and its implications for AI model development

The shift from Non-Alcoholic Fatty Liver Disease (NAFLD) to Steatotic Liver Disease (SLD) and the subcategories, Metabolic Dysfunction-Associated Steatotic Liver Disease (MASLD) and MetALD, is an important step towards a positive, metabolism-based definition of the disease. This new classification has better clinical relevance and makes the disease classification easier in research and clinical use.

The updated diagnostic criteria also have significant ramifications for the development of AI models. Most of the current AI models were trained on a set of labeled data using the past definition of NAFLD, which may result in label-shift scenarios when employed in a population based on the definition for MASLD. Furthermore, popular collections and institutional biobanks might have to be re-annotated in a retrospective fashion to comply with the new diagnostic criteria.

Future AI studies should clearly indicate whether they used NAFLD, MASLD, SLD or MetALD definitions either when building AI models or in their validation. Clear identification and understanding of disease nomenclature and labeling criteria will increase interoperability of data, help to improve external validation, and increase the reliability and generalizability of AI systems in a variety of clinical contexts (1).

### 1.2 Literature search strategy

This review was guided by PRISMA-ScR guidelines. A comprehensive literature search was conducted in PubMed, Scopus, Web of Science, and IEEE Xplore databases for articles published from 2020 to 2026. The search was updated during manuscript revision (July 2026) to identify newly published studies that satisfied the inclusion criteria. A combination of controlled vocabulary (MeSH terms where applicable) and free-text keywords related to “MASLD/NAFLD”, “artificial intelligence,” “machine learning,” “deep learning,” “imaging,” and “multi-omics” were used as search terms with the help of Boolean operators (AND, OR). The complete search strategies for each database are provided in Supplementary Table S1 to ensure reproducibility.

**Figure 1:**
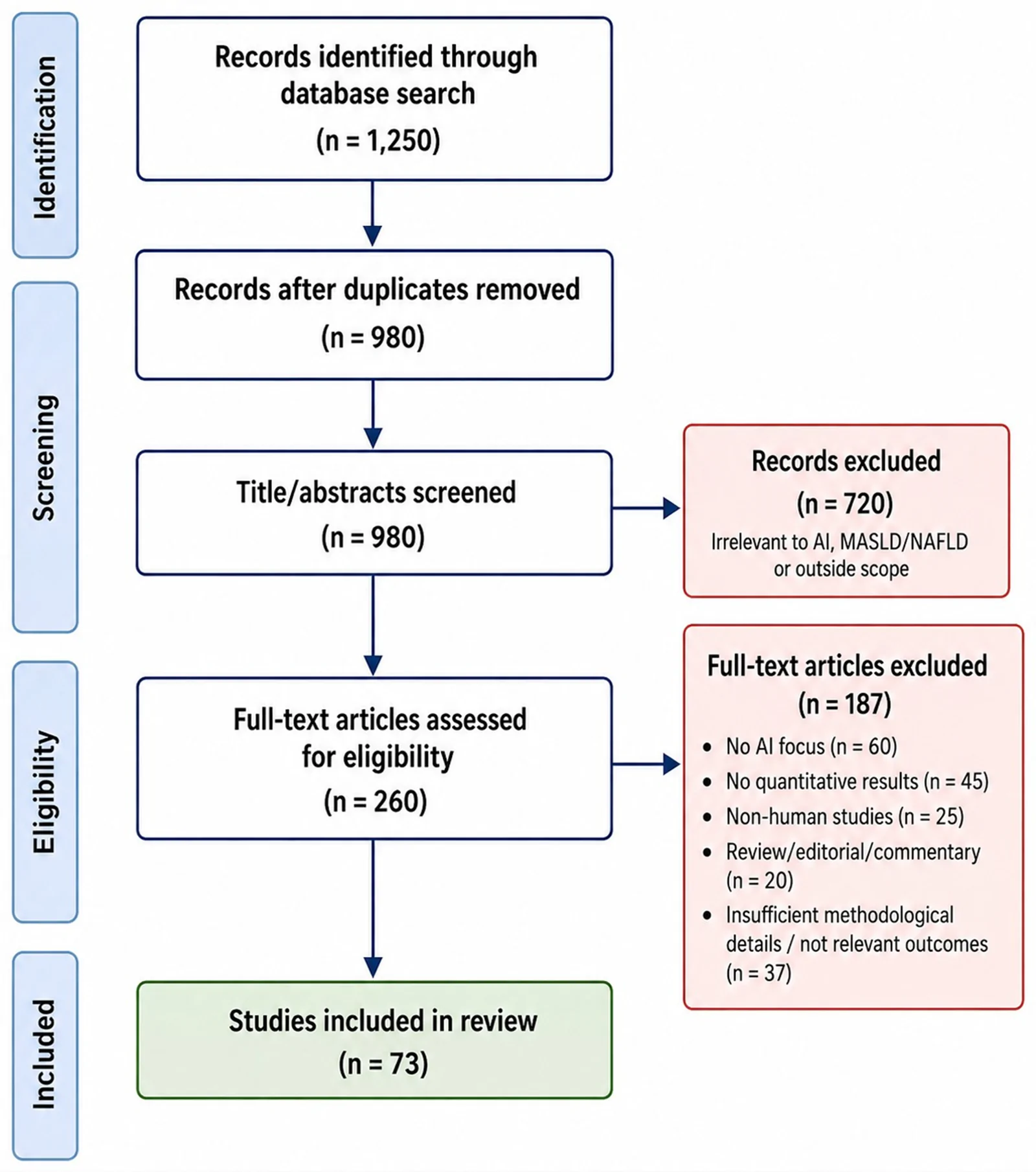
PRISMA-ScR flow diagram illustrating the literature search and study selection process.

All references that we have identified were included in the reference management system and duplicate references have been eliminated using reference management software, followed by manual verification. Two reviewers independently evaluated the title and abstract of the articles, followed by the entire paper review. If any disagreements between reviewers were resolved through discussion and consensus. Both independent reviewers conducted the screening of the studies for consistency purposes. However, in terms of the present study, which is based on the scoping review methodology, the inter-rater agreement is assessed qualitatively rather than using Cohen's kappa; this is a limitation of the current screening methodology, consistent with common practice in scoping reviews. Figure 1 illustrates the PRISMA-ScR flow diagram.

***Inclusion criteria****:* (i) AI/ML/DL models applied to MASLD diagnosis or prognosis, (ii) use of human-subject data, and (iii) relevance to at least one of three domains imaging, clinical, or multi-omics.

***Exclusion criteria****:* Studies with MetALD were considered only if the focus of study remained on AI assessment of MASLD; studies that were entirely focused on alcohol related liver illness were excluded.

The literature search yielded a total of 1,250 records from all databases searched. After removing duplicate records (270 records), 980 articles were left for title and abstract screening. After screening, 260 full-text articles were assessed for eligibility, of which 187 were excluded because they had objectives not relevant to the current study, lack of methodological information, studies that did not include humans, or studies that did not assess diagnostic or prognostic ability of AI. Eventually, 73 studies that satisfied the eligibility criteria were included in the qualitative analysis.

### 1.3 Quantitative overview of the included literature

A quantity of 73 studies published from 2020 to 2026 were used in this review. The studies concerned themselves with the use of AI in MASLD in the field of Imaging, clinical and multi-omics studies. The models were assessed with measures like AUROC, accuracy, sensitivity, specificity, Dice Similarity Coefficient, and F1 score. Overall, the reviewed studies suggest that there is great potential for the use of AI in MASLD diagnostics but call for harmonization of validation methods, standardization in reporting, and validation of clinical impact. The following sections provide a detailed discussion of these studies according to their methodological approaches and clinical applications.

This review serves the following objectives:

- To consolidate the current landscape of AI applications in MASLD diagnosis and prognosis across imaging, clinical, and omics domains.
- Comparing strategies such as CNNs, GANs, U-Nets, and ensemble learning frameworks on performance, interpretability, and real-world applicability.
- To evaluate model validation, bias, and deployment feasibility.
- To identify open challenges and gaps in data, generalizability, and clinical integration.
- To propose technically feasible and clinically relevant directions for future research.

The COVID-19 pandemic has emphasized the weaknesses in chronic disease screening and catalyzed the demand for viable AI healthcare solutions (9). The rise of MASLD concurring with the increase in obesity and type 2 diabetes needs more integrated AI systems for accurate diagnosis and risk evaluation (10). This review summarizes the recent developments in imaging, clinical and multi-omics AI to give a coherent view of the processes of MASLD diagnosis and prognosis.

Moreover, interpretability, regulatory compliance, and real-world deployment challenges remain largely unaddressed in prior literature. A technically high-performing model that cannot explain its decision logic to a hepatologist or fails to adapt across institutions is of limited clinical utility. This review evaluates such aspects by examining not just sensitivity or accuracy, but also fairness, reproducibility, computational cost, and integration potential into existing electronic health record (EHR) ecosystems (11, 12). Ethical aspects including algorithmic bias due to underrepresented populations in training data are also touched upon, especially relevant in global deployments.

In curating this review, we also acknowledge and differentiate from existing literature. For instance, while previous reviews have explored radiomics in hepatic imaging (13) and have assessed single-modality ML algorithms (14), few have provided a comprehensive a multi-axis evaluation spanning AI architecture, input types (imaging, clinical, omics), application stages (diagnosis vs. prognosis), and readiness levels (research vs. real-world). By offering structured cross-comparisons and summarizing real-time deployment examples (where available), this review aims to provide an integrated synthesis of current AI methodologies and identify future research priorities for their effective clinical translation in MASLD.

Importantly, we do not assume the reader to be an expert in both hepatology and AI. This review includes necessary context on MASLD pathophysiology, clinical workflows, and common AI terminology striking a balance between domain rigor and accessibility. Readers from biomedical backgrounds will gain a clearer understanding of what AI can realistically offer, while AI researchers can better appreciate the nuanced clinical constraints and evaluation metrics relevant to liver disease modeling. To maintain focus and ensure interpretability, this review summarizes contemporary research focused on human subjects regarding the diagnosis and prognosis of MASLD using AI methods in imaging, clinical and multi-omics areas. It also addresses the issues and recent accomplishments concerning explainable AI, multi-modal data fusion, and federated learning, and discuss their place in assisting healthcare professionals in their clinical practice.

This paper is structured as follows: Section 2 highlights the key clinical and diagnostic challenges that motivate the need for AI tools. Section 3 presents recent advancements in AI applications for imaging-based diagnosis. Section 4 explores clinical and multi-omics modeling approaches. Section 5 offers comparative insights and deployment considerations. Section 6 speaks about the Critical analysis of limitations and opportunities. Section 7 concludes with accomplishments and proposes future research and translational pathways.

## 2 Clinical and diagnostic challenges in MASLD

MASLD represents a broad clinical spectrum encompassing simple steatosis, MASH, fibrosis, cirrhosis, and HCC. This progression is gradual, clinically silent, and often undetected until irreversible damage occurs. While MASLD is widely prevalent, affecting approximately 33.6% (95% CI: 28.1%–39.5%) of the global adult population (2), it remains significantly underdiagnosed due to limitations in both clinical recognition and diagnostic modalities (15).

MASLD's clinical challenge begins with its asymptomatic nature in the early stages. Most patients remain unaware of liver dysfunction until it progresses to fibrosis or cirrhosis. This prolonged asymptomatic phase contributes to the 'silent burden' of fatty liver disease. Even when liver abnormalities are suspected, overlapping symptoms with other metabolic conditions complicate timely diagnosis, particularly in primary care settings (16).

**Figure 2:**
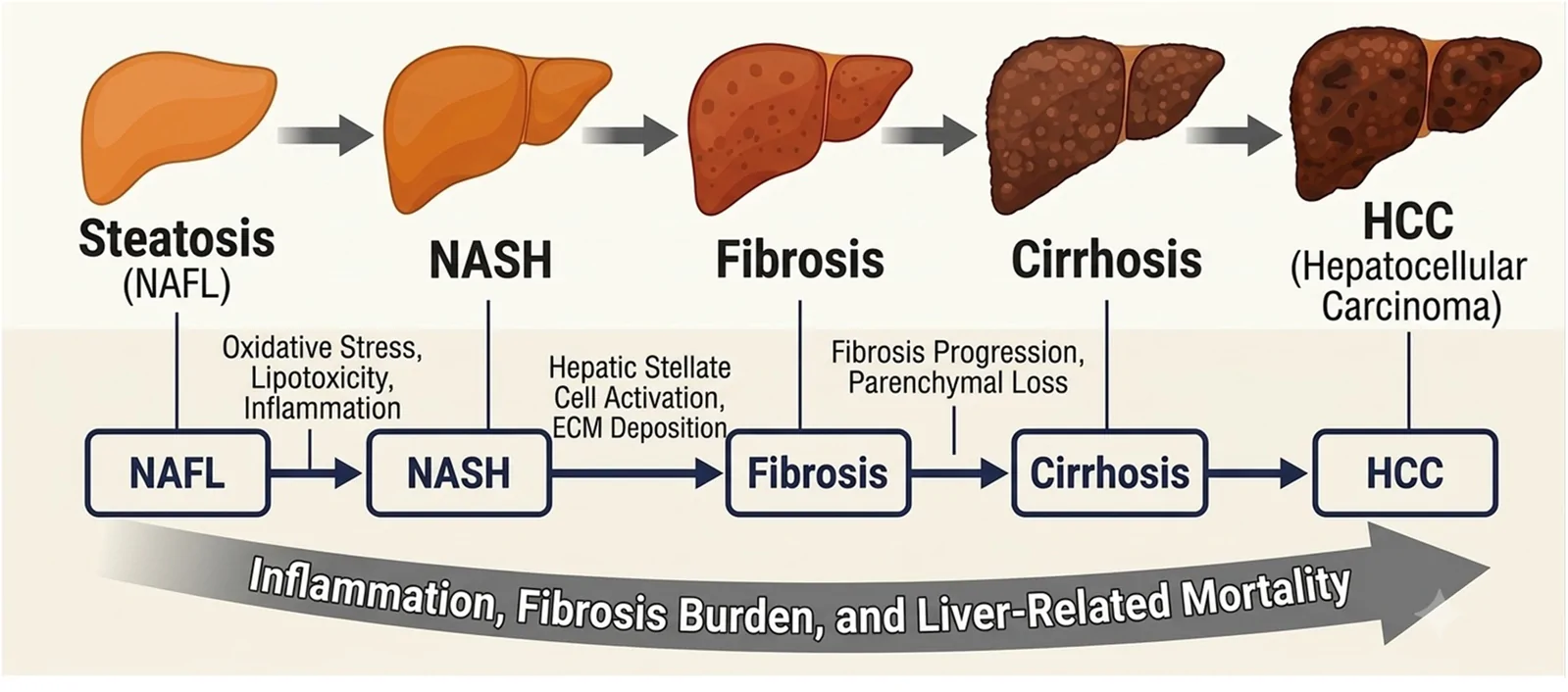
Clinical progression and pathophysiological mechanisms of MASLD from hepatic steatosis to HCC.

The natural disease course involves a continuum beginning with hepatic steatosis and potentially evolving into MASH, characterized by lobular inflammation and hepatocellular ballooning. Fibrosis and cirrhosis may follow, with or without symptoms. However, no definitive clinical markers currently predict individual disease trajectory with high accuracy. The dynamic between metabolic stress, genetics, environmental triggers, and gut-liver axis interactions makes standardization of risk assessment elusive (17). Figure 2 illustrates Clinical progression and pathophysiological mechanisms of MASLD from hepatic steatosis to HCC.

Current diagnostic tools attempt to bridge this complexity but fall short in various ways. The traditional liver biopsy, though historically considered the gold standard, presents major drawbacks: it is invasive, carries procedural risk, has low patient acceptability, and may suffer from sampling error due to the heterogeneous nature of liver lesions (18). Its high cost and need for specialist interpretation also limit widespread use, especially in low-resource settings.

To compensate for these limitations, non-invasive modalities are widely used. Ultrasound, commonly employed for initial liver fat estimation, has limited sensitivity for detecting low-grade steatosis, generally defined as hepatic fat fraction below 30% and is subject to operator variability. CT provides higher resolution but exposes patients to radiation and lacks fibrosis staging capacity. More advanced methods like MRI-PDFF and Magnetic Resonance Elastography (MRE) offer precise fat quantification and stiffness mapping, respectively, but are expensive and inaccessible in many clinical environments (19).

Elastography techniques such as Transient Elastography (TE), implemented via Fibro Scan, provide valuable non-invasive assessment of liver stiffness and steatosis through Controlled Attenuation Parameter (CAP) scores. However, they are limited by technical constraints in obese patients, presence of ascites, and operator proficiency (20). Furthermore, interpretation of results requires standardization across populations, a challenge still unmet in diverse geographic regions.

To aid fibrosis risk stratification, several serum-based scoring systems are employed, such as the NAFLD Fibrosis Score (NFS), FIB-4 Index, AST/ALT ratio, and APRI. These scoring systems are simple to calculate, making them suitable for use in primary care. However, they have wide indeterminate zones, especially in mid-stage fibrosis, and are influenced by confounders such as thrombocytopenia, age, and inflammatory states (21). Crucially, none can distinguish steatosis from MASH with high certainty. Table 1 presents a comparative overview of conventional and AI-based diagnostic modalities for MASLD, highlighting their clinical roles, strengths, limitations, and key pitfalls.

**Table 1:** Comparison of conventional and AI-based diagnostic modalities for MASLD.

Modality	Clinical role	Strengths	Limitations	Key pitfalls
Liver Biopsy	Gold standard for confirming steatosis, inflammation, and fibrosis (3, 18)	High diagnostic accuracy with detailed histopathological assessment	Invasive, expensive, risk of complications, unsuitable for routine or repeated monitoring	Sampling variability, inter-observer variability, and low patient acceptance
Ultrasound (US)	First-line imaging modality for detecting hepatic steatosis (4, 6, 20, 27)	Non-invasive, widely available, inexpensive, real-time imaging	Reduced sensitivity for mild steatosis, operator-dependent, limited quantitative assessment	Diagnostic performance affected by obesity, operator expertise, and image quality
CT Scan	Quantitative assessment of liver fat in selected clinical settings (13, 29)	Better fat quantification than ultrasound, high spatial resolution	Exposure to ionizing radiation and limited sensitivity for mild steatosis or early fibrosis	Not recommended for routine screening because of radiation exposure
MRI-PDFF/MRE	Non-invasive quantification of hepatic fat and fibrosis; reference standard in research (19, 26, 67)	High sensitivity, specificity, and quantitative assessment	High cost, limited accessibility, longer examination time	Limited availability in routine clinical practice, particularly in low-resource settings
FIB-4/NFS/APRI	Non-invasive fibrosis risk stratification using serum biomarkers (5, 21, 22)	Simple, inexpensive, easy to calculate	Moderate diagnostic accuracy and limited specificity for intermediate fibrosis	Influenced by age, metabolic comorbidities, and inflammatory conditions, resulting in indeterminate results
Transient Elastography (TE)	Non-invasive assessment of liver stiffness and steatosis using CAP scores (19–21, 26)	Rapid, repeatable, non-invasive fibrosis assessment	Reduced accuracy in obese patients and early fibrosis	Performance affected by obesity, ascites, and operator expertise
AI-Based DL/ML Models	AI-assisted diagnosis, staging, prognosis, and clinical decision support using imaging, clinical, and multi-modal data (11, 14, 32, 51, 59, 64–66, 70)	High diagnostic accuracy, scalable analysis, integration of multimodal data	Limited external validation, dataset heterogeneity, interpretability issues, and deployment challenges	Risk of algorithmic bias, poor generalizability, lack of standardized validation, and limited clinical adoption

The information presented in Table 1 was synthesized from the reviewed studies to compare the clinical roles, strengths, limitations, and key pitfalls of conventional and AI-based diagnostic modalities for MASLD.

Adding to the diagnostic dilemma is the infrequency of elevated liver enzymes in MASLD. Recent studies report that a significant proportion of patients with advanced fibrosis or MASH have normal ALT/AST levels, leading to misclassification and clinical inertia. This is particularly problematic in primary care, where abnormal liver enzymes often trigger further work-up, but their absence delays attention (22).

Ethnicity and gender also play significant roles in disease manifestation and diagnostic accuracy. For instance, Hispanic populations exhibit higher rates of hepatic fat accumulation and disease severity, while African Americans often present with lower hepatic fat despite metabolic risk factors. Women, especially post-menopausal, may exhibit accelerated fibrosis progression. Yet, most diagnostic algorithms do not incorporate such demographic modifiers, resulting in systemic underestimation or overestimation of disease burden (23).

Furthermore, comorbid conditions such as T2DM, PCOS, and obesity frequently confound biochemical profiles and non-invasive assessments. Insulin resistance and hyperlipidemia alter baseline markers are used in fibrosis scores. For example, a diabetic patient may have elevated AST, and low platelet counts unrelated to liver pathology, skewing risk scores like FIB-4 (24).

Another layer of complexity is introduced by insufficient training and awareness at the primary care level. Surveys indicate that many general practitioners lack the confidence or tools to screen and monitor patients for MASLD. Liver ultrasound may be skipped due to resource constraints or lack of referral protocols. Even when scores such as FIB-4 are available, they may not be integrated into electronic health records or clinical decision support systems (25).

Access disparities compound the problem. In developing regions, elastography and MRI are often confined to urban tertiary centers. Many healthcare systems do not reimburse non-invasive diagnostics unless advanced liver disease is suspected, creating a perverse incentive to wait until symptoms appear. These barriers further delay diagnosis and reduce the window for effective intervention (26).

Finally, although AI-based tools are increasingly proposed to address diagnostic inefficiencies, they are only as reliable as the input data and annotation standards used. Models trained in poorly labeled, demographically skewed, or modality-inconsistent data may perpetuate biases and reduce trust in AI systems among clinicians. The promise of AI in hepatology will only be realized if upstream diagnostic quality is ensured.

In summary, the diagnosis of MASLD is hindered by a fragmented landscape of invasive and non-invasive tools, misaligned clinical awareness, socio-demographic variability, and infrastructure limitations. Tackling these barriers is essential for effective treatment, early intervention, and trustworthy deployment of AI-enhanced solutions.

Despite the fact that conventional diagnostic methods are still highly relevant in clinical practice, the limitations of these methods have motivated the advent of novel AI-assisted technologies for improving the accuracy of diagnosis, disease staging, and prognosis. The next section discusses the recent developments in the technological field of imaging analysis aimed at the diagnosis of MASLD.

## 3 AI in imaging-based diagnosis of MASLD

The diagnostic complexity of MASLD stems from its heterogeneous presentation and progressive nature. While conventional imaging modalities like US, CT, and MRI are pivotal for initial detection, their sensitivity and specificity vary widely depending on disease severity. AI, especially DL, is reshaping the MASLD imaging landscape by offering enhanced precision, standardization, and automated decision support.

Recent advances in deep neural networks have enabled accurate detection and quantification of hepatic steatosis, fibrosis, and inflammation reducing the limitations associated with manual image interpretation. This section explores how AI enhances imaging modalities for MASLD diagnosis, focusing on CNNs, GANs, U-Nets, and transformer-based models, and examining current benchmarks, explainability frameworks, and existing datasets.

### 3.1 AI-powered imaging modalities in MASLD

**Figure 3:**
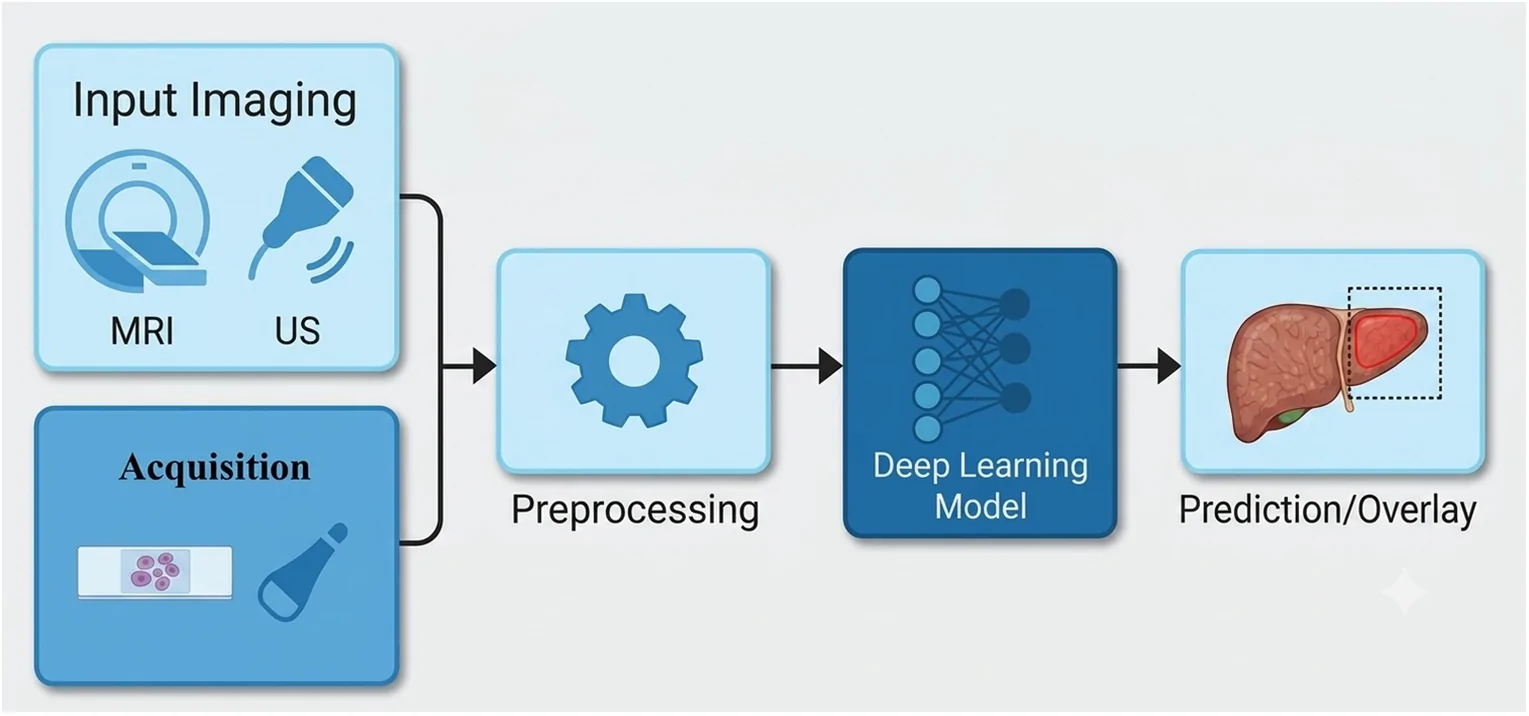
Deep learning pipeline for imaging-based MASLD diagnosis.

**Figure 4:**
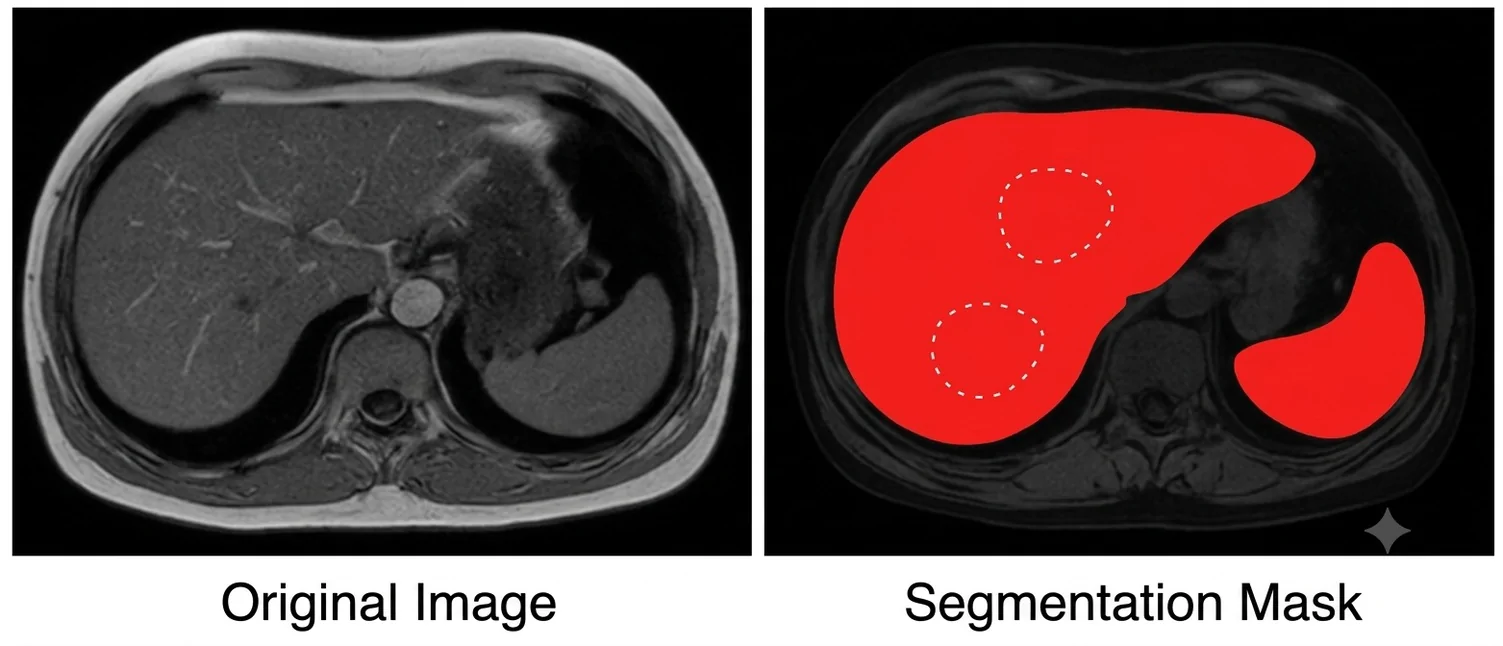
AI-aided liver segmentation using MRI.

Among imaging techniques, ultrasound remains the most accessible first-line modality but suffers from limited sensitivity for detecting mild or early-stage steatosis, particularly when hepatic fat content is below 20%. Recent CNN-based models applied to imaging data have shown pooled sensitivity of 91% (95% CI: 84%–95%) and specificity of 92% (95% CI: 86%–96%), with an AUC of 0.97, demonstrating diagnostic performance comparable to that of experienced radiologists and conventional reference standards (27). CNNs trained on 2D B-mode scans extract echotexture and attenuation-based features imperceptible to human observers. Figure 3 presents the deep learning pipeline for imaging-based MASLD diagnosis and Figure 4 illustrates AI-aided liver segmentation using MRI.

The models developed using DL technology for MRI segmentation have been able to obtain Dice Similarity Coefficient values ranging from 0.88 to 0.96 in the majority of reported implementations, achieving high liver segmentation accuracy.

AI-based segmentation models like U-Net and ResUNet++ are used to automatically delineate hepatic regions, minimizing operator dependency and improving repeatability. These models are increasingly integrated with multi-parametric data (PDFF, T2*, MRE) to provide comprehensive fibrosis staging (28).

CT scans, while less preferred due to radiation, are still used in surgical and oncological settings. Researchers have demonstrated CNNs that detect textural liver signatures from low-dose CT to predict fibrosis stage. Some studies have applied generative adversarial networks (GANs) to create synthetic examples of rare conditions such as bridging fibrosis, which helps mitigate class imbalance in limited datasets (29). Table 2 provides Comparative summary of representative image-based AI Models for MASLD diagnosis. In order to ensure the transparent comparison, the studies are compared based on the imaging modality, the size of the sample, study design, methods of validation, and evaluation metrics reported.

**Table 2:** Comparative summary of representative image-based AI models for MASLD diagnosis.

Modality	AI model	Clinical task	Sample size	Study design	Validation strategy	Performance	95% CI
Ultrasound (27)	CNN (2D)	Steatosis classification	∼500	Retrospective	Internal validation	AUC: 0.97	(95% CI: 0.95–0.98)
MRI (28)	U-Net variant	Liver segmentation and fibrosis assessment	∼1,000	Multicenter retrospective	Internal validation	Dice > 0.90; AUROC ∼0.95	NR
CT (29)	CNN + GAN	Fibrosis prediction	∼700	Retrospective	Internal validation	AUROC: 0.88–0.90	NR
MRI/MRE (32)	Swin Transformer	Fibrosis staging	∼800	Retrospective	Internal validation	AUROC: 0.94–0.96	(95% CI: 0.78–0.88)

NR, Not Reported.

Although AUROC and Dice score were most frequently reported metrics of evaluation, the majority of studies reported no calibration measures (i.e., Brier score or calibration slope) or details about the balance of the evaluated classes, which restricts the transparency of comparison of evaluations in terms of calibration and clinical applicability of the model.

### 3.2 Datasets and annotation challenges

AI model performance is inherently tied to the quality and quantity of data. Public datasets remain scarce in MASLD imaging, unlike fields like retinal or lung imaging. Some of the widely used datasets include the UK Biobank Liver MRI and proprietary hospital collections.

However, these datasets often vary in image resolution, scanner types, ethnic diversity, and labeling protocols. Manual annotations for liver boundaries and fibrosis staging introduce subjectivity, which can bias AI training. Multicenter collaborations and federated learning are now being adopted to improve generalizability (30). The datasets used in the below discussion were taken from the studies cited in this review given the fact that these datasets are commonly used in AI-based researches for MASLD and share specific features such as public availability (where applicable), dataset quality, indicator of thorough annotation, and relevance to the liver imaging issues of segmentation, steatosis detection, and fibrosis evaluation. Table 3 summarizes the commonly used public and private datasets in AI-based imaging studies for MASLD. The datasets summarized in Table 3 were extracted from the reviewed studies, and the corresponding source references are indicated within the table.

**Table 3:** Summary of representative public and private datasets used in AI-based imaging studies for MASLD.

Dataset Name	Type	Modality	Approx. Size	Annotation	References
UK Biobank Liver MRI	Public	MRI-PDFF	>32,000 participants	Automated MRI-PDFF measurements with expert quality control	(25)
Standard T2-weighted MRI Dataset	Private (Multicenter Clinical Dataset)	MRI	∼1,000 subjects	Expert fibrosis annotations	(28)
FibroScan-labelled Ultrasound Dataset	Hybrid (Private + Public)	Ultrasound	∼2,644 images (224 patients)	FibroScan-assisted expert labelling	(32)
Quantitative Ultrasound Dataset	Private (Institutional)	Ultrasound	∼500 patients	Expert radiologist annotations using quantitative ultrasound	(27)
Multi-view Ultrasound Dataset	Private (Institutional)	Ultrasound	Several hundred patients	Radiologist-confirmed steatosis labels	(4)
CT Radiomics Dataset	Private (Institutional)	CT	Several hundred subjects	Histology-based hepatic steatosis labels	(13)

The quality of annotation is the key factor in determining the performance of AI technologies used in MASLD imaging. Inaccurate, inconsistent, or incomplete annotations result in the presence of label noise that negatively affects feature learning, segmentation accuracy, and generalization ability of deep learning models. Inter-observer variability and intra-observer variability are particularly important for liver segmentation, steatosis grading, and fibrosis scoring. In order to increase the reliability of annotations, standardization, expert consensus, quality assurance processes, and validation by several experts have been recommended by scholars. As a result, the availability of annotated datasets from different institutions is the necessary factor in reliable AI systems for MASLD diagnostics and prognosis (31).

### 3.3 XAI for MASLD imaging

**Figure 5:**
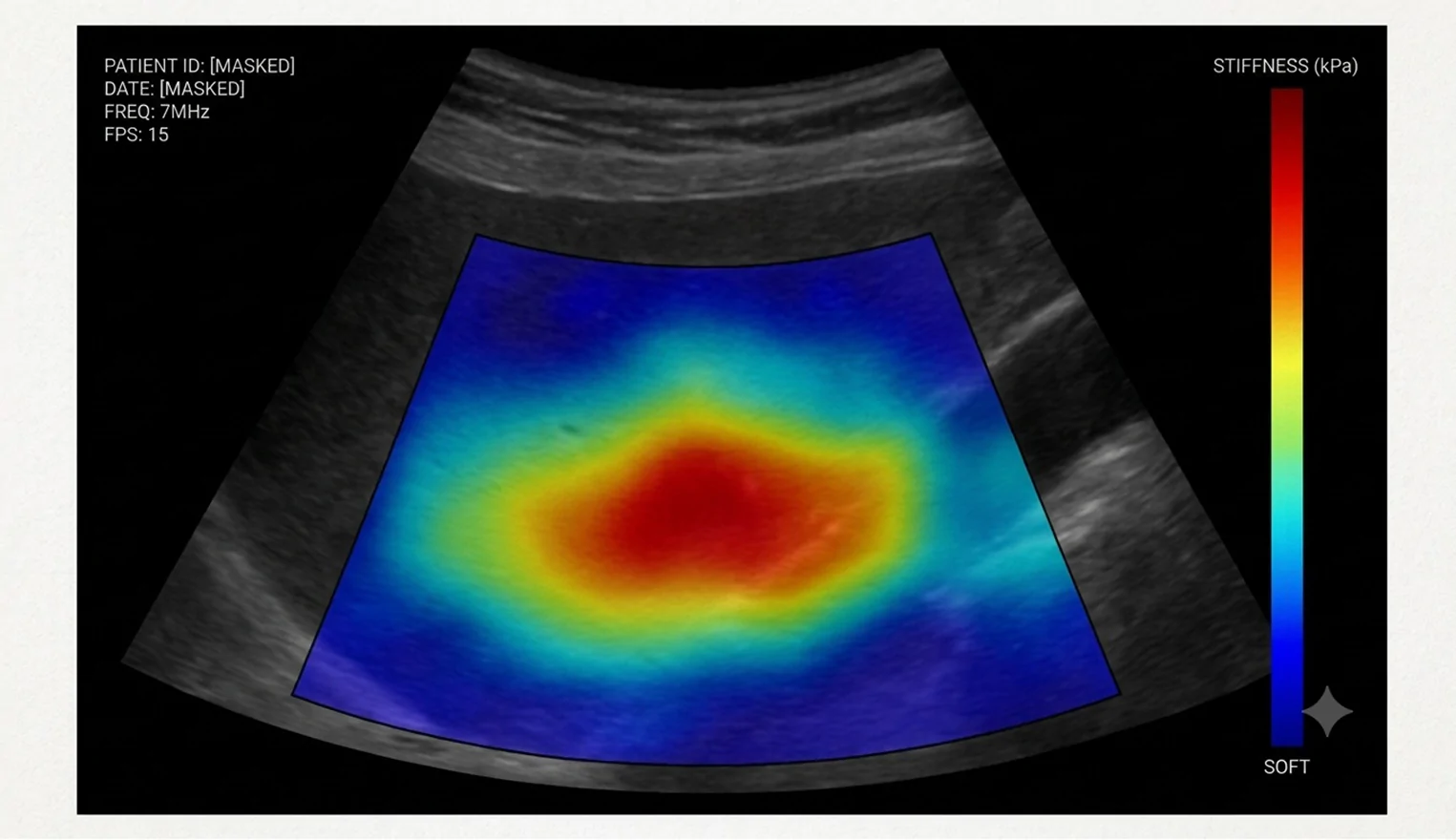
Grad-CAM overlay on liver ultrasound images for XAI based MASLD diagnosis.

The black-box nature of DL models remains a significant barrier in clinical settings. Clinicians demand transparency especially when AI decisions contradict their judgment. Tools like Grad-CAM, SHAP, and Integrated Gradients are now integrated into imaging pipelines to generate saliency overlays on liver images (32). Figure 5 presents the Grad-CAM overlay of liver ultrasound images for XAI-based MASLD diagnosis.

Saliency maps are supposed to enable clinicians to place their trust in the system, although it is unknown whether they encourage genuine calibrated trust or just give people increased confidence without regard to whether the prediction is correct, and these overlays can detect spurious correlations; for instance, sometimes models depend on spurious signals such as shadowing of ribs and boundaries of the diaphragm.

### 3.4 Emerging trends and novel contributions

Modern MASLD imaging models are moving toward multi-task learning, where the network jointly predicts steatosis, fibrosis, and even inflammation scores from a single input. Transformer-based models like Swin-UNet and Vision Transformers (ViTs) are gaining traction for their superior context modeling and generalization ability (33).

**Figure 6:**
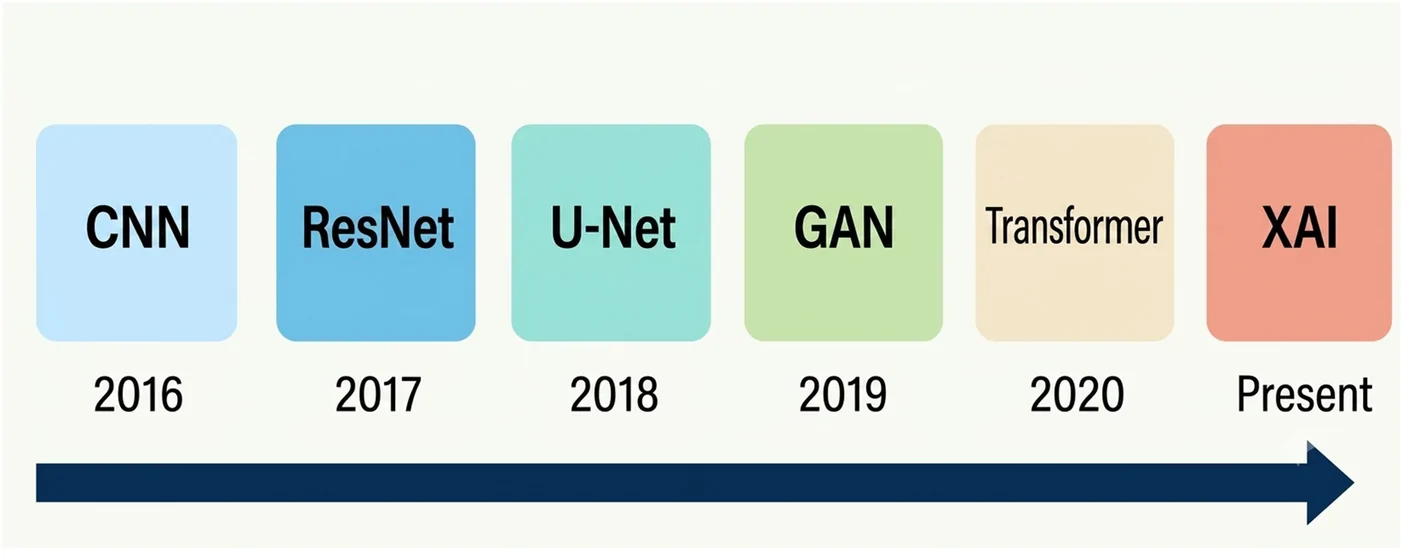
Evolution of AI architectures in MASLD imaging.

Another emerging paradigm is self-supervised learning, which pre-trains models on unlabeled liver scans using contrastive methods before fine-tuning on small, labeled datasets, a major advantage in data-scarce hepatology (34). In a recent study, Delfan and others have introduced a non-invasive method for diagnosing steatosis using a machine learning model that combines information-fusion techniques. In contrast to single-model methods, this approach is able to use features from various sources in its cascade model, resulting in a more efficient classification of diseases. This innovation creates a synergy with the growing interest toward hybrid AI systems to use various pieces of information for better diagnostic efficiency (35). Figure 6 illustrates the evolution of AI architectures in MASLD imaging, and Table 4 summarizes the benefits and barriers of AI across different imaging modalities for MASLD.

**Table 4:** Benefits and barriers of AI by imaging modality.

Modality	AI benefits	Key barriers
Ultrasound	Real-time aid, standardization	Low resolution, operator dependence
MRI	Quantification, segmentation, fibrosis map	High cost, availability
MRE	Stiffness prediction, fibrosis staging	Motion sensitivity, limited access
CT	Texture analysis, 3D modeling	Radiation risk, lower adoption in hepatology

### 3.5 Limitations and future scope

Despite promising results have been obtained, challenges still remain. The challenges specific to imaging, dataset, low cross-site generalizability, a lack of prospective validation, and barriers to the integration of clinical workflow will be discussed in detail together with their clinical and multi-omics counterparts in Section 6.

## 4 ML models using clinical and multi-omics data

Understanding the complex etiology and progression of MASLD necessitates integrative modeling frameworks that go beyond imaging. Recent advances in ML and multi-omics data integration have opened new avenues for detecting disease earlier, stratifying patient risk, and predicting progression toward MASH, fibrosis, and HCC. This section synthesizes how classical and modern AI approaches leverage structured clinical data, electronic health records (EHR), and high-throughput multi-omics to transform MASLD diagnostics and prognostics.

### 4.1 Machine learning using structured clinical and EHR data

**Figure 7:**
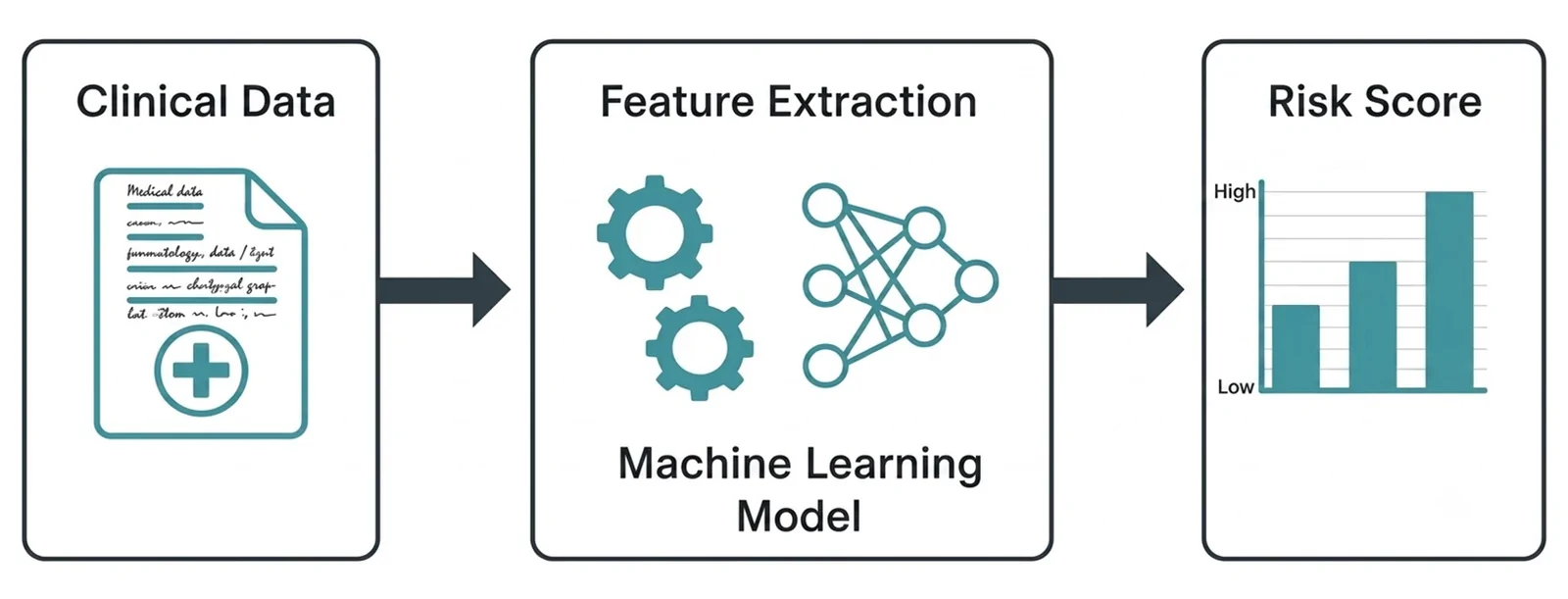
ML pipeline from clinical data to risk score.

Conventional MASLD risk models such as the NAFLD Fibrosis Score, FIB-4, or APRI use linear regression over limited biomarkers. In contrast, ML models trained on structured datasets (e.g., lab panels, vitals, demographics, comorbidities) exploit non-linear relationships to enhance prediction accuracy. Random Forest, XGBoost, and Support Vector Machine (SVM) models have demonstrated AUROC values in the range of 0.82 to 0.91 in the context of fibrosis staging and NAFLD predictions based on clinical data (36, 37). Table 5 presents various ML models used for clinical MASLD risk stratification and Figure 7 shows the ML pipeline from clinical data to risk score.

**Table 5:** ML models using clinical variables for MASLD risk stratification.

Model	Features	Target	AUROC	Dataset	95% CI
Random Forest	BMI, AST, ALT, HbA1c, GGT, triglycerides	Fibrosis (F ≥ 2)	0.84	NHANES + Biopsy-verified	NR
XGBoost	12 metabolic & liver biomarkers	Steatosis (>33%)	0.88	UK Biobank	NR
Logistic LASSO	Age, diabetes status, HDL, ALT, hypertension	NASH vs. NAFL	0.83	Korean NHI Registry	NR

NR, Not Reported.

Temporal models trained on EHR data (RNNs, Transformers) provide additional insights by learning disease trajectories over time (38).

### 4.2 ML applications in genomic and epigenomic risk prediction

The genomic landscape of MASLD is characterized by key polymorphisms: PNPLA3 (rs738409), TM6SF2 (rs58542926), and MBOAT7 variants. ML models trained on genotyping arrays can stratify patients based on inherited predisposition to steatohepatitis or advanced fibrosis (39).

**Figure 8:**
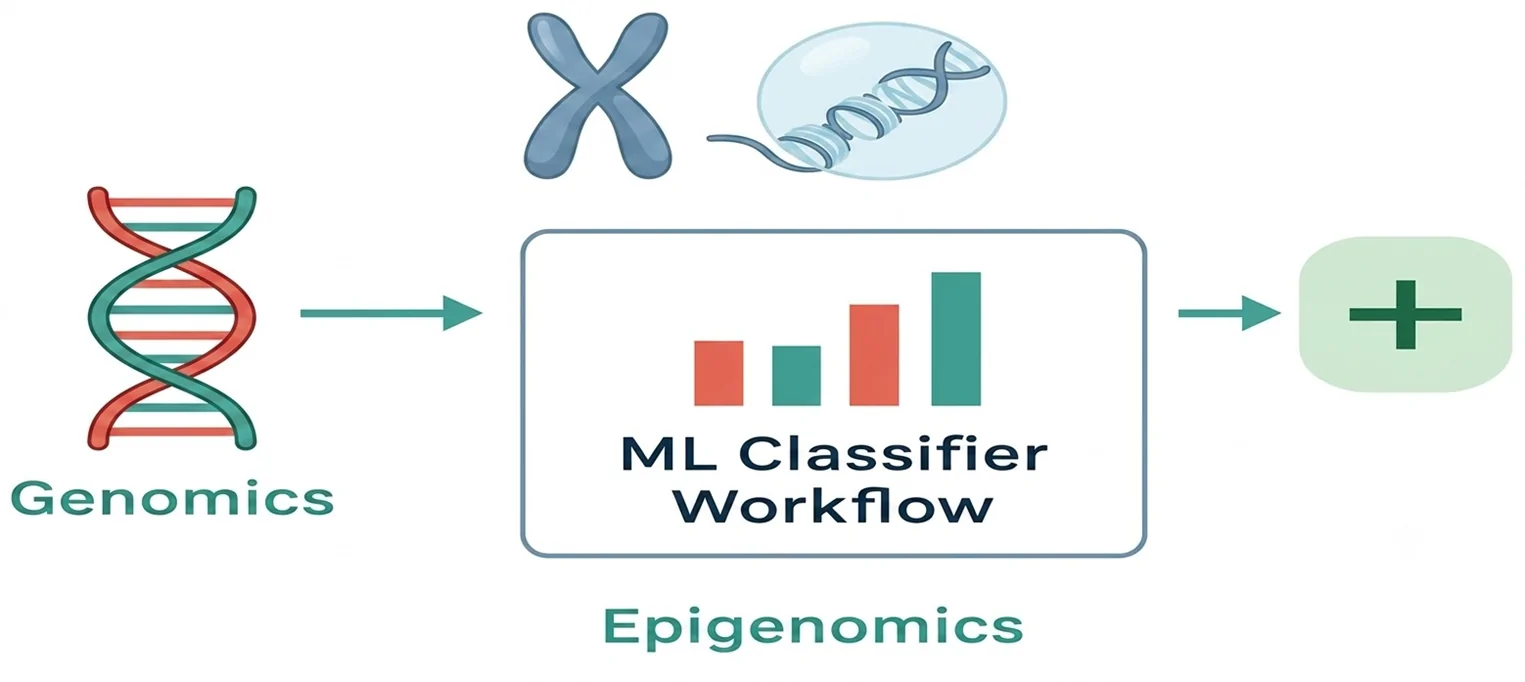
Genomic and epigenomic feature workflow for ML-based MASLD stratification.

SNP-based classifiers using SVMs or Gradient Boosting Trees have shown predictive capabilities with AUROC > 0.80. The integration of epigenomic markers such as DNA methylation patterns in liver biopsies further improves staging accuracy, especially when combined with transcriptomic data (40). Table 6 presents the ML-Based MASLD Studies used in Genetic and Epigenetic Predictors and Figure 8 illustrates Genomic and Epigenomic feature workflow for ML-Based MASLD Stratification.

**Table 6:** Genetic and epigenetic predictors used in ML-based MASLD studies.

Omics layer	Key markers	Use case	ML model	Performance	95% CI
Genomics	PNPLA3, TM6SF2, MBOAT7	Risk stratification	XGBoost	AUROC 0.81	NR
Epigenomics	Methylation in COL1A1, TGF*β*	Fibrosis stage classification	Elastic Net	AUROC 0.79	NR
					NR

NR, Not Reported.

### 4.3 Transcriptomic and proteomic signatures in MASLD

**Figure 9:**
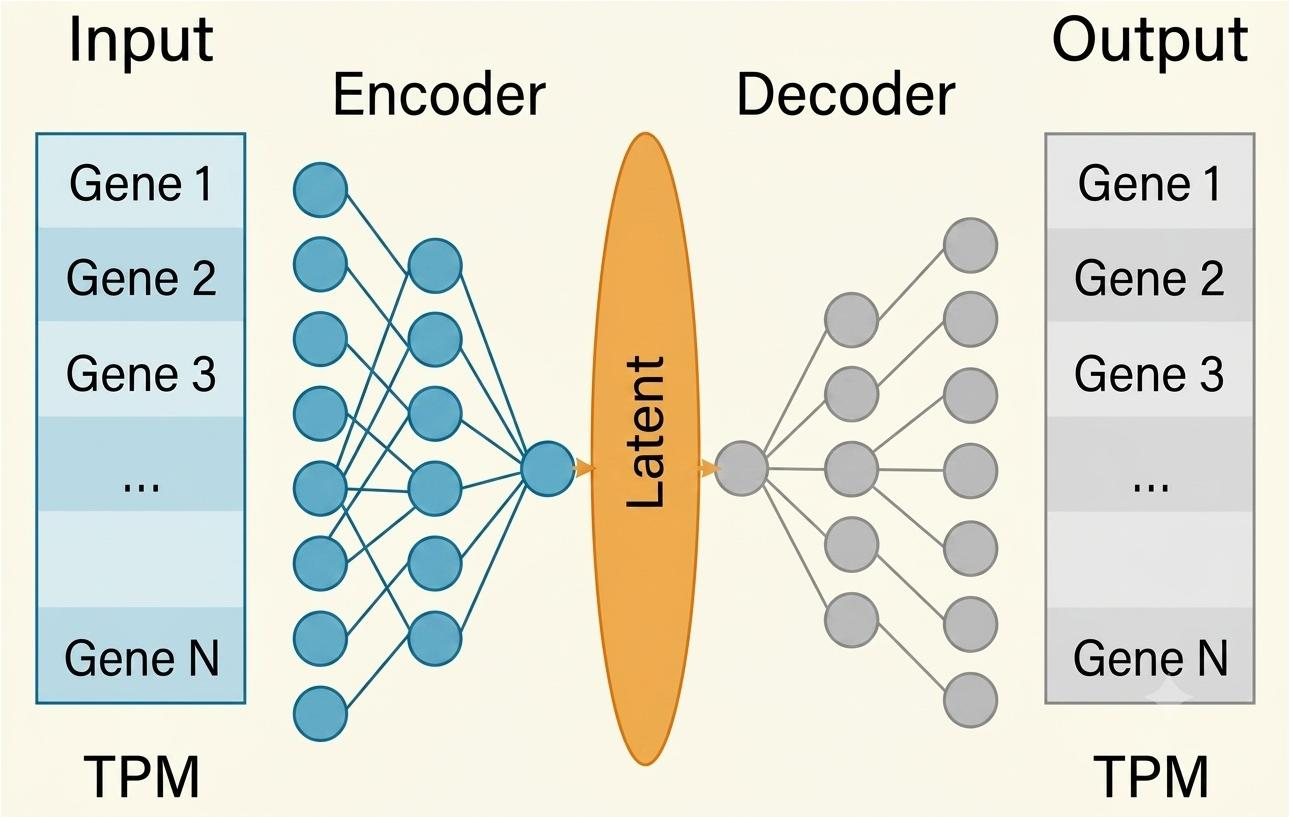
Autoencoder-based compression of liver transcriptomic profiles.

Transcriptomics from liver biopsy samples have revealed key inflammatory and fibro genic markers. Differentially expressed genes (DEGs) like IL6, TNF*α*, COL1A1, and MMP9 are upregulated in MASH and cirrhotic stages. Recent DL models have applied autoencoders and multi-view learning to extract embeddings from transcriptomic and proteomic profiles, enabling MASLD subtyping (41). Figure 9 shows the Compression of liver transcriptomic profiles using autoencoders.

Proteomic analyses using mass spectrometry (MS) further contribute to stratifying NAFL vs. MASH cases. Predictive pipelines using RF and LightGBM on proteomics datasets from plasma samples have shown promising classification performance for distinguishing disease subtypes (42).

### 4.4 Metabolomic and microbiome-driven ML models

**Figure 10:**
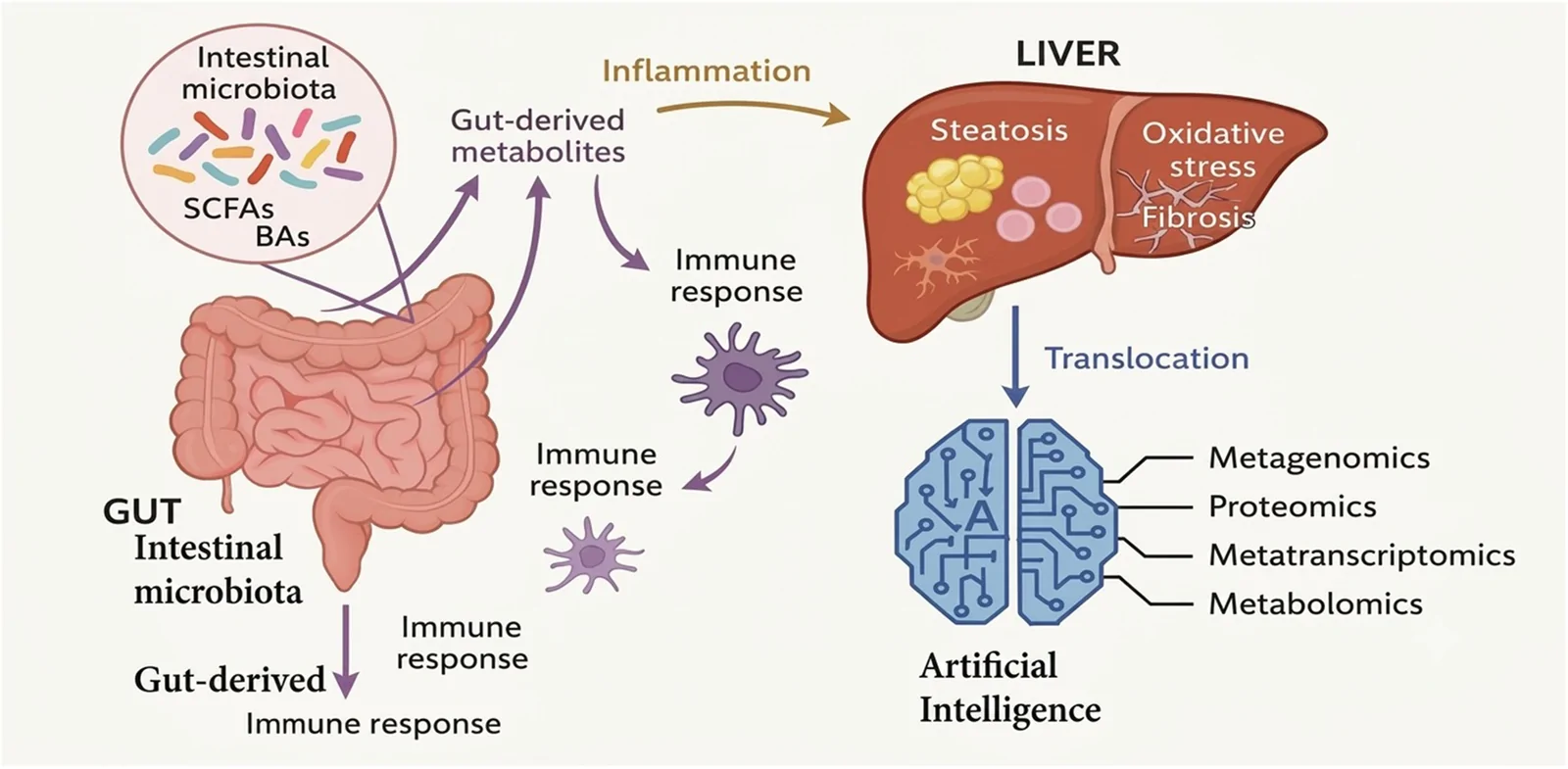
Gut-liver axis in MASLD with AI integration.

Metabolomic signatures particularly altered bile acids, branched-chain amino acids (BCAAs), and lipid species have been widely studied. Using mass spectrometry, researchers have developed MASLD staging models with metabolite panels and achieved AUROC scores exceeding 0.90 for MASH detection (43). In the gut microbiome, metagenomic sequencing reveals compositional shifts in taxa (e.g., Bacteroides, Firmicutes) in MASLD. ML classifiers such as Naïve Bayes, RF, and DL-based autoencoders can predict disease class using microbial abundances, suggesting a systems-level link between gut dysbiosis and liver inflammation (44). Metabolomic and Microbiome Data in MASLD using ML models is shown in Table 7 and Gut-Liver Axis in MASLD with AI Integration shown in Figure 10.

**Table 7:** ML models using metabolomic and microbiome data in MASLD.

Data Type	Features	Model	Target	AUROC	95% CI
Metabolomics	Bile acids, ketones, fatty acid esters	Random Forest	NASH diagnosis	0.91	NR
Microbiome	120 bacterial taxa (16S rRNA)	Gradient Boosting	Steatosis class	0.87	NR

NR, Not Reported.

### 4.5 Data fusion, causal models, and multi-omics integration

The challenge in multi-omics ML for MASLD is integrating heterogeneous data types while maintaining interpretability. Recent studies apply graph neural networks (GNNs) and transformer-based fusion architectures that simultaneously learn from transcriptomics, proteomics, and clinical EHR (45).

**Figure 11:**
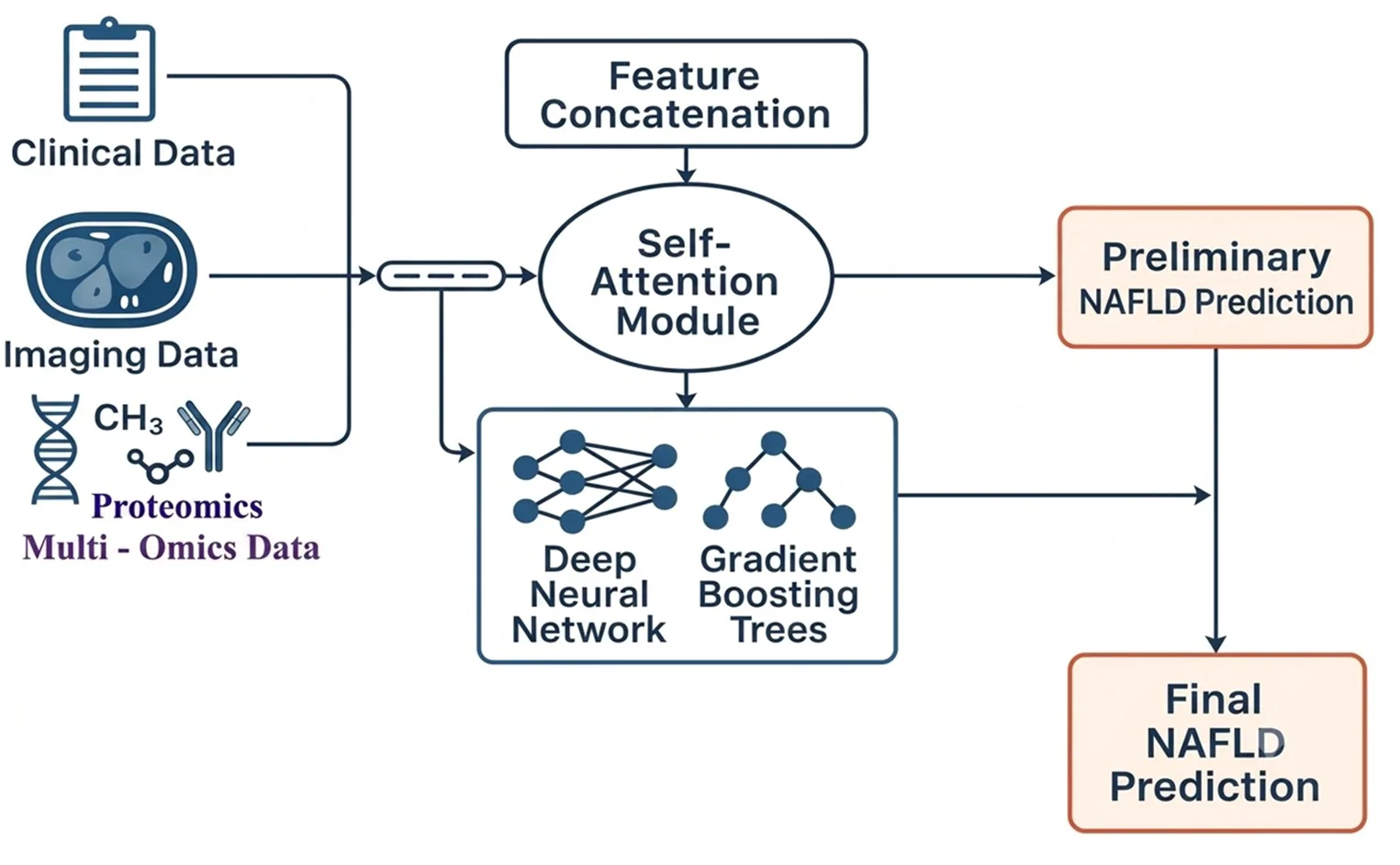
Hybrid data fusion architecture for multi-modal MASLD prediction.

In one study, MOFA2 (Multi-Omics Factor Analysis) fused liver histology, transcriptome, and metabolomics data to identify latent phenotypic clusters in MASLD patients (46). Causal modeling using Do Why or causal forests is also gaining attention to distinguish correlation from true biological causality in MASLD markers. Federated learning frameworks like FedBioMed are emerging for cross-center collaboration without sharing raw omics data, preserving privacy while training robust models (47). Apart from the use of framework development, recent research is looking into the use of federated learning for AI-based privacy protection in the field of liver disease and related services. The results of these studies indicate that using federated learning will allow institutions to collaborate on their AI models while ensuring that patient data remain within their own organizations. This shows that data security can be improved through FL, and so can the usefulness of developed models in the future (48). Table 8 presents FL studies related to AI in the diagnosis of MASLD and their key findings regarding the preservation of data privacy during AI application in multicenter data processing. Figure 11 represents a schematic Hybrid data fusion architecture for Multi-Modal MASLD Prediction.

**Table 8:** Federated learning approaches relevant to AI-based MASLD diagnosis.

References	Study type	AI task	FL contribution	Relevance to MASLD
(30)	Original research	NASH stage classification from distributed imaging datasets	Federated General Visual Representation Learning for NASH stage classification	Direct evidence demonstrating FL for MASH/MASLD classification.
(52)	Review	Medical imaging and healthcare AI	FL for medical applications	Provides the technical foundation supporting future multi-center MASLD AI systems.
(57)	Review	Healthcare informatics	FL in healthcare informatics	Supports adoption of FL for privacy-preserving MASLD diagnosis across hospitals.

Despite the power of AI in multi-omics analysis, clinical translation is limited by interpretability, generalizability, and data bias. To address this, XAI tools like SHAP, LIME, and attention visualizations are increasingly embedded into MASLD risk models. The aim of using these tools is to ensure model predictions that are personalized for each patient, in order to build clinical trust; however, empirical evidence on whether saliency-based explanations reliably improve appropriate clinician trust, as opposed to causing some sort of dependence on false model forecasts, is contradictory. Moreover, reproducibility remains a challenge. Most studies are trained on single-center, Western cohorts. Efforts are underway to establish global federated multi-omics databases across Asia, Europe, and South America to improve generalizability.

The information fusion framework proposed by Delfan et al. indicates how cascade ensemble learning architecture is used to integrate data-level and feature-level information. Though this review takes into account the bigger picture of integrating magnetic resonance imaging results, clinical information, and multi-omics data for the evaluation of MASLD, the cascade information fusion principle employed by the authors is an example of successful combination of different information sources leading to better classification result.

#### 4.5.1 Challenges in multi-omics data integration

Although the advent of multi-omics integration has many applications in the diagnosis and prognosis of MASLD, there are many technical challenges that must be overcome. Thus, it is common for multi-omics databases to include a huge pool of molecular features while having a small sample size, which poses a great risk of model overfitting, unstable poor model transferability.

Multi-omics AI research is often plagued by the “large p, small n” problem, which refers to the relationship between a high number of molecular features (high dimensionality, p) and and a low number of observations (small sample size, n); this imbalance substantially increases the risk of overfitting. In this regard, studies mentioned above primarily utilized various regularization techniques (LASSO, Elastic Net, Recursive Feature Elimination), combined with some dimensionality-reduction methods (Principal Component Analysis, autoencoders, Multi-Omics Factor Analysis). Notably, only a few works employed any form of external validation, suggesting that the overall claims of improvements should be considered with caution until proven through larger multi-site trials (49).

The cascade learning model proposed by Delfan et al. is a different ensemble-based method for the evaluation of steatosis stages without the need for an invasive study. Unlike regular single-model designs, this model combines additional information using the cascade learning approach, which improves the quality of diagnostics. However, in line with other studies that have been analyzed here, this one used mainly internal cross-validation to measure the performance, with little external validation and calibration check performed. Thus, there is a need for further validation of cascade learning models in different clinical centers.

## 5 Comparative insights, benchmarks, and deployment considerations

AI models for MASLD span a rich spectrum of data modalities and architectures, yet their real-world impact hinges not just on accuracy but on their clinical relevance, interpretability, adaptability, and integration readiness. In this section, we provide an in-depth comparative analysis of state-of-the-art AI systems for MASLD, explore the nuances of benchmarking practices, examine deployment challenges, and offer a critique of the current landscape in both academic and clinical contexts.

### 5.1 Evaluating AI paradigms across modalities

**Figure 12:**
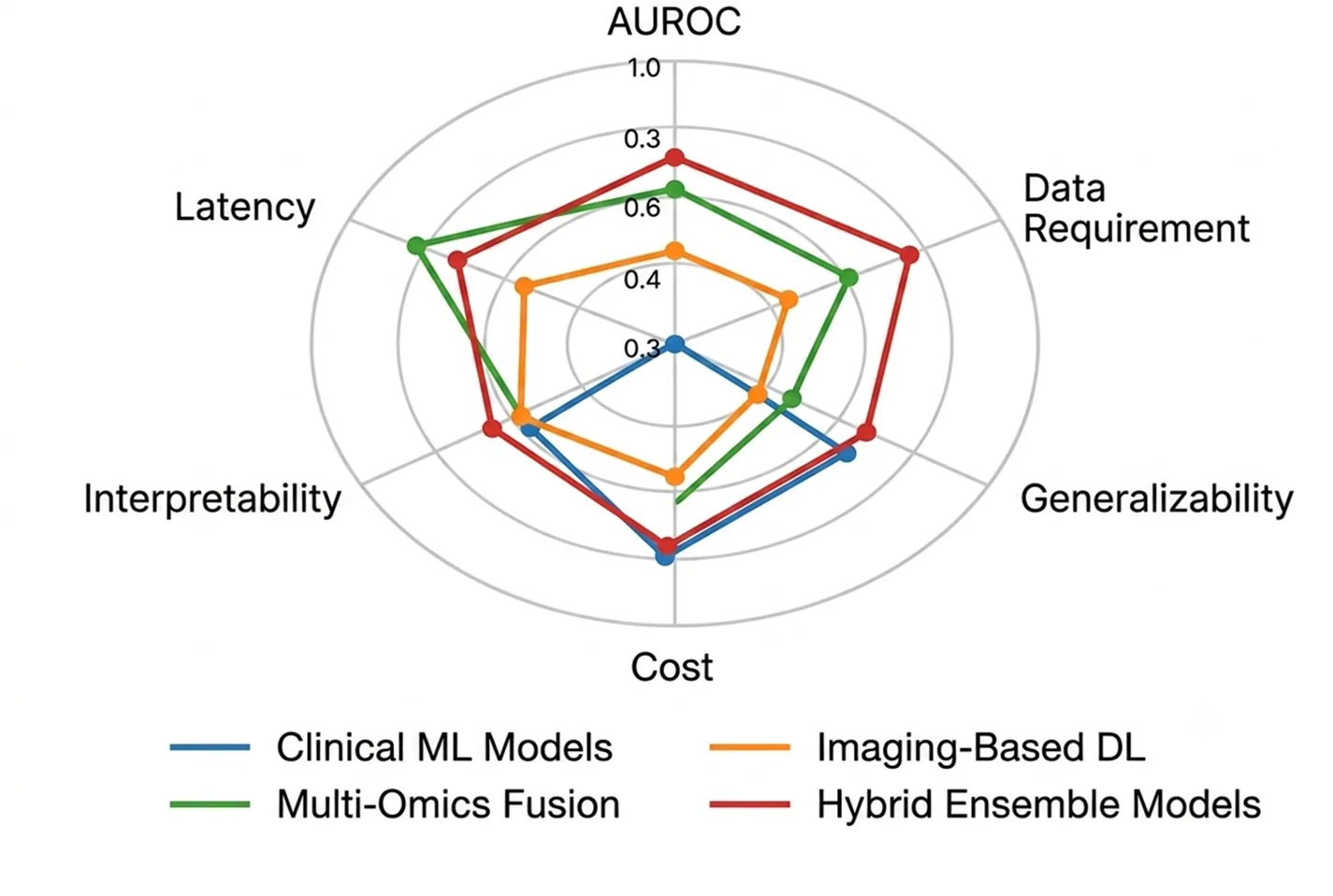
Comparative visualization of AI modalities in MASLD.

AI systems for MASLD range from classical ML models using structured EHR to DL architectures operating on imaging, transcriptomic, or multi-omics data. Each paradigm exhibits strengths and limitations depending on the context of application. The Comparative Strengths and Limitations of AI architectures for MASLD are shown in Table 9, Figure 12 Visualize comparison of AI Modalities in MASLD.

**Table 9:** Comparative strengths and limitations of AI architectures for MASLD.

Architecture	Strengths	Limitations
CNN	High spatial precision for image classification and detection; extensively validated in medical imaging	Requires large annotated datasets and has limited capability for modelling long-range dependencies
U-Net	Excellent pixel-level segmentation performance with preservation of spatial information through skip connections	Computationally intensive and sensitive to class imbalance during segmentation
GAN	Generates synthetic images to address class imbalance and supports image-to-image translation	Training instability and potential generation of unrealistic artifacts
Transformer (ViT/Swin-UNet)	Superior long-range feature modelling with strong cross-domain generalization	Requires large datasets and high computational resources; limited interpretability
Ensemble Models	Improved predictive accuracy and robust generalization by combining multiple models	Increased computational complexity, reduced explainability, and challenging clinical deployment

The majority of the studies that were analyzed revealed that imaging-related deep learning algorithms tended to have AUROC values ranging between 0.85 and 0.95, and liver segmentation models based on MRI have Dice Similarity Coefficient values not less than 0.90. As for the AUROC values of clinical models, they varied from 0.80 to 0.91, while the combined results of clinical data and imaging outperformed the results conferring the advantages of AI using different forms of data (50).

### 5.2 Beyond AUROC: multidimensional benchmarking for trustworthy AI

Traditional metrics such as AUROC, accuracy, and F1-score fail to capture deployment readiness. To move beyond lab-based efficacy, AI models must also be assessed for:

- Latency and runtime efficiency
- Memory footprint and hardware requirements
- Explainability and interpretability metrics
- Robustness under adversarial or noisy conditions

Multidimensional Performance Metrics of Leading AI Models shown in Table 10. Many studies fail to report latency and generalizability yet claim state-of-the-art performance. The benchmarking ecosystem lacks transparency datasets that are reused without documenting preprocessing pipelines, and comparison baselines are not standardized (51, 52). Benchmarking efforts in this space would also benefit from alignment with established AI reporting guidelines TRIPOD + AI for prediction models (50), CLAIM for medical imaging AI (53), DECIDE-AI for early-stage clinical evaluation (54), and CONSORT-AI/SPIRIT-AI for clinical trials (55, 56).

**Table 10:** Multidimensional performance metrics of leading AI models.

Model	AUROC	Latency (ms)	Memory (MB)	Explainability tool	External validation	95% CI
CNN (Liver Ultrasound)	0.92	125	88	Grad-CAM	No	NR
XGBoost (EHR Features)	0.87	30	10	SHAP	Yes	NR
Transformer (Histopathology)	0.93	230	190	Attention Map	No	NR
Multi-Omics Stacked Ensemble	0.96	380	280	SHAP + Permutation Test	No	NR

NR, Not Reported.

In our opinion, upcoming studies focused on AI for the diagnosis of MASLD ought to consider combining AUROC, along with other parameters that will describe prediction accuracy as well as utility. Providing information about calibration and testing performance under practical decision thresholds would provide necessary clarity and ease translation into everyday clinical hepatology practice (57).

When evaluating various AI models for MASLD, external validation and model generalizability are also important factors that need to be noted. Multiple studies have focused on internal validation but there are very few studies that have utilized external validation on independent cohorts. As a result, performance metrics claimed might not be representative of the actual clinical performance in the real world because of differences in imaging techniques, scanner types, prevalence of disease, and patient characteristics. Future research should therefore focus on multicenter external validation, prospective clinical verification, and standardized testing using different populations (58, 59).

### 5.3 Explainability, trust, and clinical utility

**Figure 13:**
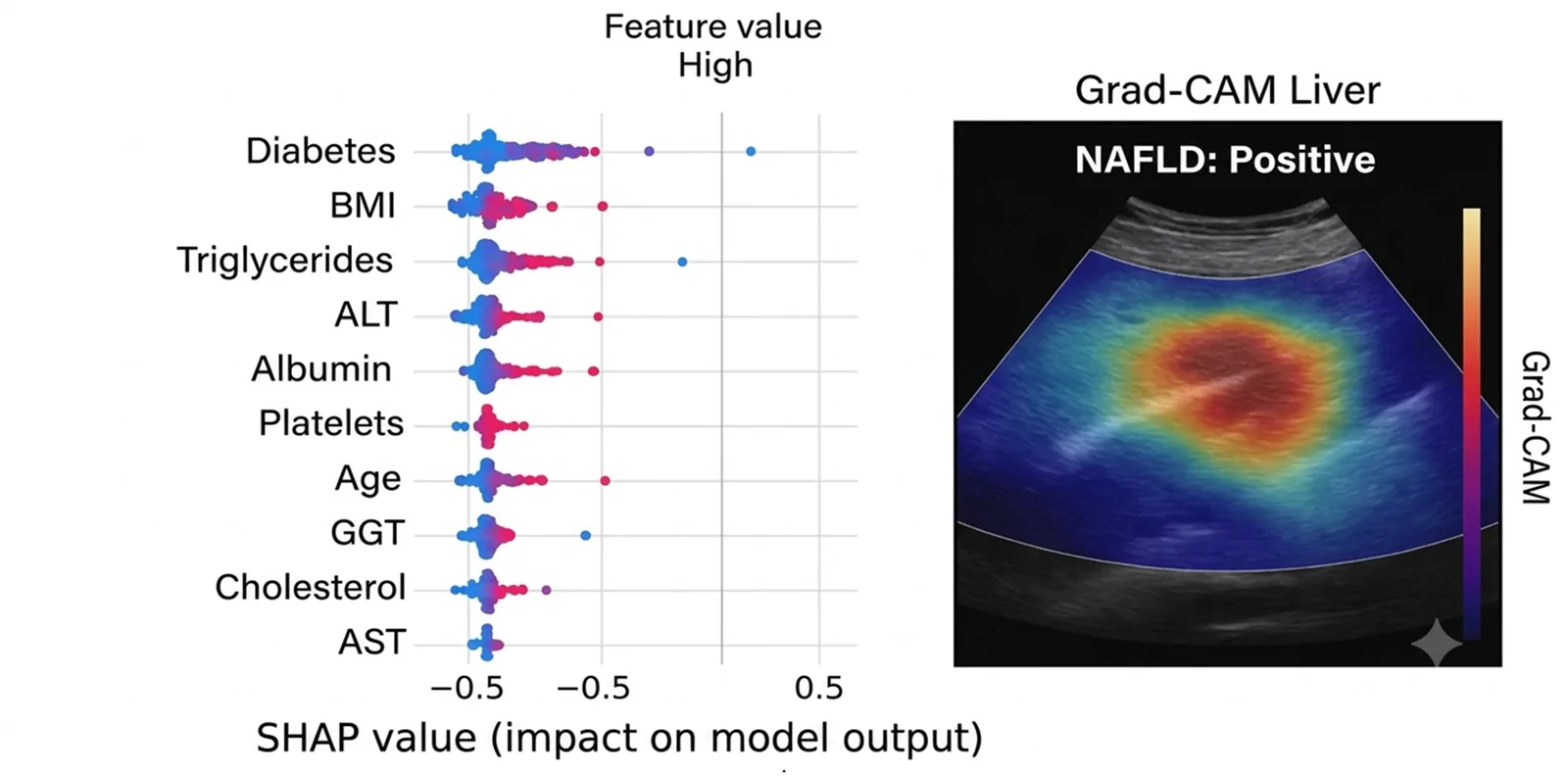
Integrated XAI dashboard for MASLD predictions.

Model interpretability remains a cornerstone for clinical acceptance. XAI tools such as SHAP, LIME, and Grad-CAM provide *post hoc* visual or tabular attributions of feature influence. Integrated XAI Dashboard for MASLD Predictions is shown in Figure 13.

However, a critical issue is over-reliance on *post hoc* explanations, which often conflict across methods. For instance, Grad-CAM may highlight irrelevant liver zones while SHAP suggests liver enzymes as top predictors, leading to clinician distrust. Many AI systems use XAI more as a cosmetic patch rather than embedding explainability into model design. Only a few models (e.g., attention-based transformers) offer intrinsic interpretability. There is a need for model-first XAI frameworks, especially in high-stakes diagnoses (60, 61).

### 5.4 Deployment realities: From cloud to bedside

**Figure 14:**
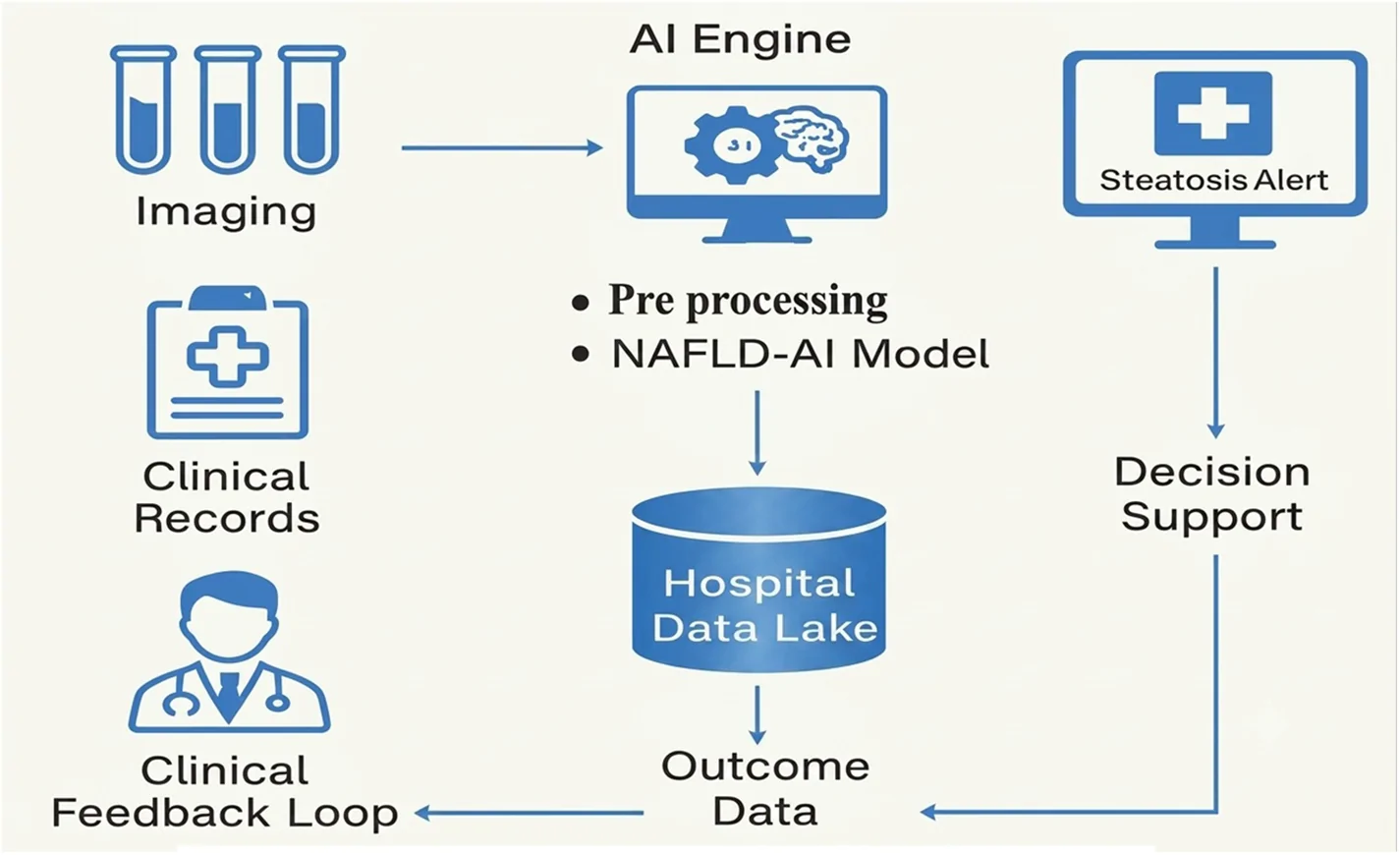
End-to-end clinical deployment architecture for MASLD-AI.

Figure 14 represents End-to-End Clinical Deployment Architecture for MASLD-AI Despite the surge in high-performing AI contexts, few have translated into bedside tools. Deployment challenges include:

- **EHR Integration:** Compatibility with FHIR or HL7 formats is rare
- **Regulatory Approval:** Most models lack FDA/CE compliance documentation
- **Hardware Deployment:** Edge deployment of low-power devices remains underexplored
- **Interoperability:** Models trained on private datasets perform poorly in public repositories

Many models neglect practical deployment issues, such as balancing speed with accuracy or ensuring interpretability without adding complexity. They also rarely consider how difficult it is for clinicians to change established workflows.

For successful clinical translation of AI tools for MASLD, one needs to get their technology integrated into healthcare agencies rather than operate as a separate diagnostic tool. The AI models capability of working together with PACS, EHRs, and RIS systems will allow for automatic image retrieval as well as qualitative reporting and data loss. Moreover, the rollout process should strictly follow the rules of applicable regulations and should ensure compliance with the patients' data security (62).

### 5.5 Data bias, generalizability, and ethical gaps

AI models show reduced performance in ethnic subgroups, pediatric populations, and non-obese patients with MASLD. These biases arise due to skewed training data or the use of proxy variables like BMI, which may not generalize. There is a persistent lack of fairness audits, sensitivity analyses, and ethical oversight in AI publications. Moreover, federated learning remains mostly academic, with limited trials in hepatology departments due to infrastructure and policy constraints (63, 64).

### 5.6 Longitudinal vs. cross-sectional AI models: temporal trade-offs

AI models for MASLD diagnosis rely on cross-sectional data static snapshots of clinical or imaging inputs failing to leverage the temporal dynamics of disease progression. Longitudinal models, in contrast, use patient histories across time points, offering insights into how NAFL progresses to MASH, fibrosis, and beyond. The Comparison between cross-sectional and longitudinal AI Models for MASLD is shown in Table 11.

**Table 11:** Comparison between cross-sectional and longitudinal AI models for MASLD.

Aspect	Cross-sectional models	Longitudinal models
Data Input	Single visit/sample	Multiple visits/samples
Modeling Techniques	Random Forest, CNN, SVM	RNN, LSTM, Temporal CNN
Progression Modeling	No	Yes
Deployment Readiness	High (simpler input)	Medium (complex data requirements)
Interpretability	Easier	Challenging due to sequence dependencies

While longitudinal models like Bi-LSTM or Transformer variants offer progression tracking, their need for dense and regular follow-up data makes them impractical for under-resourced clinics or retrospective analyses (65).

### 5.7 Reproducibility, auditability, and model drift in clinical AI

A recurring concern in MASLD AI literature is reproducibility. Models trained on one cohort often fail when validated externally. Additionally, few works provide open code, standardized preprocessing scripts, or model cards with a transparency gap that limits clinical translation.

***Key Issues:***

- **Lack of Model Cards**: Insufficient reporting of model architecture, hyperparameters, and implementation details limits reproducibility and independent validation
- **No Audit Logs**: Deployment trials rarely include logging mechanisms for clinician-AI disagreement tracking
- **Concept Drift**: Shifts in population health (e.g., post-COVID metabolic changes) may erode performance

Though awareness of reproducibility requirements continues to grow, many published AI models for MASLD remain difficult to reproduce due to inadequate reporting of preprocessing procedures, training protocols, hyperparameter values, and the availability of source code. Implementing standardized reporting practices while increasing the use of transparent documentation, model cards, version control tools, and reproducible machine learning processes would improve transparency, make it easier for independent validators, and speed up the adoption of AI-based diagnostic systems (66, 67).

### 5.8 Interpretability vs. clinical trust: the XAI tradeoff

Building upon the explainability methods discussed in Section 5.3, this subsection focuses on the gap between algorithmic explanations and clinician trust in real-world practice. For instance, a SHAP plot might highlight platelet count as a key factor, while the hepatologist focuses on ALT/AST trends. Moreover, these tools offer no causal guarantees.

***Open Problems:***

- Disagreement in feature importance across tools
- No confidence intervals in explanations
- Missing standards for interpretability quality assessment

Apart from providing visual explanations, methods of explainability must be assessed with the help of some reliable quality criteria. Crucial evaluation criteria include faithfulness, which shows the faithfulness of the explanation to the model's inner reasoning; stability, which shows how the explanation remains consistent with small perturbations; robustness, and finally, clinical concordance, which shows that the AI explanation corresponds to the medical judgment of a hepatologist. The use of these criteria for evaluation purposes in upcoming MASLD studies will promote clarity and the confidence of physicians and ease the introduction of explainable AI systems into practice.

The Human-in-the-Loop (HITL) model represents a promising path for the eventual clinical use of AI systems in MASLD diagnosis. Instead of replacing doctors, AI systems can help radiologists and hepatologists with quantitative assessments and predictions such as lesion localization, stage of fibrosis, but without credibility without validation by physicians. Doctors always bear the responsibility for AI results, solving ambiguous cases, and applying history and laboratory tests to the final diagnosis. Collaborative methods would be able to combine the technological efficiency of AI and medical professionals' experience improving diagnostic reliability, decreasing automation bias, and increasing clinician trust in routine practice.

Although *post-hoc* explanation techniques including Grad-CAM, SHAP, and LIME have gained popularity, there are limitations to these approaches. Different explanation methods produce different importance scores for the same set of input features based on their diverse explanation mechanisms, which leads to inconsistent interpretation of the same prediction. Moreover, visual heat maps may show the areas that seem to be justified from the clinical point of view but do not accurately represent the decision-making of the model. Such discrepancies may lower the level of trust for clinicians and make AI assisted diagnosis more complicated. As a result, future studies must focus on development of the common evaluation framework for the existing methods in terms of consistency, robustness, reproducibility, and relevance in the clinical domain (64).

While *post-hoc* explainability approaches such as Grad-CAM, SHAP, and LIME yield helpful visualizations and explanations at the feature level, they may not accurately represent the actual decision-making process employed by deep learning models. Therefore, explainability should be subjected to systematic assessment according to the criteria of faithfulness, stability, robustness, and clinical concordance, rather than simply relying on visual evaluation. Moreover, the combination of intrinsically interpretable model architectures and *post-hoc* explainability methods may enhance the transparency of AI systems and therefore improve their acceptance by clinicians and make them safer for clinical application for NAFLD diagnosis (65).

Numerous studies have emphasized the importance of interpretability only after model development, rather than being built into the design from the outset. Models should be co-developed with clinical experts using human-in-the-loop feedback to ensure that explanations are not only accurate but also meaningful. The detailed analysis of interpretability issues, deployment problems, and ethical aspects is given in Section 6.

### 5.9 Model accountability, legal liability, and ethics in deployment

A growing challenge for MASLD AI is not technical but legal and ethical:

- Who is liable if a model falsely rules out fibrosis?
- Should AI predictions override a senior hepatologist's diagnosis?
- What protocols exist for model recalls or retraction post-deployment?

Legal and Ethical Barriers for MASLD-AI Deployment in Hospitals is shown in Table 12, While performance metrics dominate most of the works, the real-world use of AI demands regulatory clarity, including GDPR/ HIPAA alignment, model recall processes, and liability sharing contracts (62, 63, 68).

**Table 12:** Legal and ethical barriers for MASLD-AI deployment in hospitals.

Barrier	Impact	Solution direction
No AI Governance Boards	Unchecked deployment	Institutional AI ethics review boards
Lack of Traceability	No logs for decision audits	Blockchain-based audit trails
Informed Consent Gaps	Patients unaware of AI usage	Transparent consent processes in EHR forms
Black-box Models	Poor clinician accountability sharing	Shift toward interpretable, certifiable AI

### 5.10 AI-driven clinical trial design and precision subtyping

AI can go beyond diagnosis as a design tool for MASLD clinical trials. By clustering patients based on omics and imaging data, trials can stratify participants better, avoiding failures due to misclassification.

**Novel Contributions in Literature:**

- DL-based clustering algorithms identify silent MASH subtypes
- Reinforcement learning helps optimize trial enrollment criteria dynamically
- Surrogate endpoints modeled via autoencoders to shorten trial duration

While promising, most AI-stratified trials remain in silico. Very few studies offer randomized control trials validating AI-based inclusion criteria, and there is little pharma-AI collaboration to date (64, 65).

### 5.11 Incremental value over existing non-invasive tests

A question that remains unaddressed in the literature reviewed is, what value does AI provide beyond low-cost options like FIB-4, NFS, and CAP at clinically relevant cutoffs (for example, FIB-4 < 1.3 low-risk/ > 2.67 high-risk). AUROC measures of clinical AI models mentioned in this review range from 0.82 to 0.91 and are not distinctly better than FIB-4/NFS in the same cohorts (5, 21, 22). One must also mention that AUROC measures alone do not indicate clinical usability, because AUROC can be the same among models with different reclassification at actionable cutoffs. An informal discussion reveals that NRI and DCA measures are hardly ever mentioned together with AUROC measures in literature. Future research should compare at least with FIB-4/NFS/CAP at the relevant thresholds and include NRI/DCA analyses to demonstrate benefits over existing instruments (69).

## 6 Critical analysis and limitations

The application of AI to the diagnostic and prognostic modeling of MASLD has demonstrated considerable promise across multiple fronts ranging from enhanced early-stage detection to high-dimensional multi-omics integration. However, despite this progress, several technical, clinical, and ethical limitations remain. This section critically analyzes the shortcomings of current AI-driven approaches and outlines key barriers to real-world deployment and trustworthiness.

### 6.1 Critical evaluation of current approaches

The studies examined in this review demonstrate performance superiority of AI models, especially deep learning architectures over traditional rule-based systems in identifying hepatic steatosis, staging fibrosis, and predicting disease progression. However, these performance gains often occur under tightly controlled experimental settings with ideal datasets, which rarely reflect the diversity, complexity, and noise present in real-world clinical practice (70).

For example, CNN-based models deployed on imaging modalities such as MRI-PDFF or ultrasound often exhibit overfitting to specific data cohorts and equipment vendors, reducing generalizability (71). Similarly, ML models trained on omics data suffer from cohort imbalance and batch effects, making them less transferable across institutions or ethnic populations (72). Table 13 below summarizes these critiques across AI categories.

**Table 13:** Critical limitations by AI category in MASLD modeling.

AI Category	Limitation	Example
Imaging (CNN, U-Net)	Vendor-specific data bias	Models fail to generalize across ultrasound machine brands
Clinical (XGBoost, RF)	Missing values, demographic confounders	Age/gender skew in training datasets
Multi-omics (Deep AE, SVM)	Lack of large, annotated datasets	Few labeled genomics datasets linked with biopsy-proven MASLD
Explainable AI (Grad-CAM, SHAP)	Clinical misinterpretation of saliency maps	Highlighted regions may not match radiologist expectations

This scoping review was not prospectively registered (e.g., via OSF), which may limit transparency of its *a priori* methods.

### 6.2 Interpretability vs. Accuracy Tradeoff

A recurring challenge in AI for MASLD lies in the trade-off between model accuracy and explainability. While deep networks like ResNet and Transformer models offer high precision, their black-box nature raises trust issues among clinicians (73). As discussed in Sections 5.3, 5.8, model interpretability remains a major challenge for clinical adoption. This discordance often leads to clinician-AI disagreements and reduces adoption rates in real-world settings.

Moreover, current explainable AI methods do not always support uncertainty quantification, which is crucial in medical decision-making. Future work must aim to bridge interpretability-performance gap without sacrificing clinical realism.

### 6.3 Clinical deployment gaps

Despite the high potential of AI-based tools, clinical deployment remains a bottleneck. Integrating AI predictions into electronic medical records (EMRs), ensuring real-time inference capability, and aligning with clinician workflows are complex engineering and regulatory challenges (74).

For instance, most AI pipelines lack feedback loops for continuous learning or clinician override, leading to stagnant or outdated models over time. Model drift and reproducibility issues are non-trivial. Moreover, no clear consensus exists on thresholds for intervention or risk stratification across institutions. For imaging-based tools specifically, deployment into PACS or radiology dashboards remains constrained by regulatory approval pathways and persistent interpretability hurdles.

### 6.4 Ethical, regulatory, and social barriers

Several social and regulatory limitations persist in the adoption of AI for MASLD:

- **Bias & Fairness:**

ML models trained predominantly on datasets from high-income Western populations may fail to perform accurately in underrepresented geographies (75).

- **Regulatory & Ethics:**

Currently, AI diagnostics are regulated by specific instruments. Thus, in the U.S., AI/ML software is classified as SaMD due to the FDA's implemented Predetermined Change Control Plan (PCCP, Dec 2024), which allows algorithmic updates without additional submissions. Most AI-based diagnostic software in the EU is classified as EU MDR IIa/IIb, and positive results from the assessment by a notified body are required. AI diagnostics pose high risks, which is activation of risk management system, accountability, oversight of AI by humans from August 2027 (76). Data sharing in federated learning is still limited by GDPR/HIPAA restrictions and the requirements of auditability/versioning are not usually satisfied (77).

Sharing of clinical or genomic data for federated training is limited due to patient confidentiality and GDPR/HIPAA regulations. Regulatory bodies also require model auditability and versioning, which many AI pipelines currently lack (77).

- **Deployment Barriers:**

Integrating AI models into existing hospital IT systems can be challenging due to incompatible workflows, legacy infrastructure, and resistance to workflow changes.

- **Data Heterogeneity:**

Clinical, imaging, and genomic data often come from multiple institutions using different formats and standards, complicating preprocessing, harmonization, and model training.

- **Lack of Prospective Trials**:

Few AI tools for MASLD imaging have been validated prospectively across centers.

- **Model Interpretability:**

Black-box AI models hinder clinician trust; transparent explanations of model predictions are critical for adoption in high-stakes decision-making.

- **Clinical Integration:**

Successful adoption requires seamless incorporation into clinical pathways, with minimal disruption to existing processes and clear evidence of benefit to patient outcomes.

### 6.5 Dataset limitations and annotation gaps

Another major shortcoming is the lack of standardized datasets. Many studies rely on private institutional repositories or imbalanced datasets, lacking sufficient labels or follow-up annotations. Particularly for fibrosis staging or progression forecasting, longitudinal data is sparse (78). Imaging-specific models often show similar imbalance, with underperformance on rare fibrosis grades or mixed steatohepatitis phenotypes due to skewed training distributions.

Public datasets such as NIDDK, UK Biobank, or TCIA have been underutilized or inconsistently labeled. Synthetic data generation and data harmonization across cohorts represent promising, but currently underdeveloped, directions.

### 6.6 Critical gaps identified

To synthesize, critical limitations in current literature and practice include:

- **Limited external validation of models**

Most published models are trained and tested on institution-specific or small public datasets without real-world multi-center validation, raising concerns on generalizability. In imaging pipelines specifically, site-specific artifacts and scanner biases frequently degrade performance when models are applied to external cohorts.

- **Lack of cross-modality harmonization between imaging and omics**

Integration of imaging biomarkers (e.g., steatosis grade) with genomics, epigenomics, or metabolomics remains underexplored and poorly standardized.

- **Poor interpretability and mismatch with clinician intuition**

Despite the use of explainability techniques like Grad-CAM and SHAP, there exists a disconnect between algorithmic saliency and domain expert judgment.

- **Unclear thresholds for deployment in clinical settings**

There is no consensus on performance benchmarks (e.g., AUROC, specificity) required for safe deployment in clinical workflows, especially for NAFLD staging.

- **Lack of fairness-aware training and representative cohorts**

Model performance often degrades in underrepresented populations (e.g., pediatric NAFLD, ethnic minorities), reflecting dataset imbalance and unaccounted sociodemographic bias.

- **Inadequate feedback loops for post-deployment refinement**

Few models include mechanisms to learn from clinical feedback or evolve with changing patient populations or diagnostic guidelines.

- **Sparse utilization of longitudinal data for disease trajectory modeling**

Most current models are cross-sectional, neglecting time-series trends critical for forecasting progression from NAFL to MASH, fibrosis, or HCC.

- **Limited robustness to real-world imaging artifacts and noise**

AI models often fail in the presence of variability in scan quality, patient motion, or inter-device differences, particularly in ultrasound and MRI-based methods.

- **Absence of cost-aware model selection and optimization**

Many proposed architectures are computationally intensive, ignoring practical constraints in edge-deployable or resource-constrained clinical environments.

- **Minimal incorporation of comorbid condition modeling**

Co-occurring conditions like T2DM, obesity, or cardiovascular risk are not explicitly modeled in most prediction pipelines, reducing clinical relevance.

- **Data silos and lack of interoperable AI frameworks**

There is no unified infrastructure for integrating multi-center EHRs, imaging archives, and biobank data in a privacy-preserving, federated setting.

- **Limited use of uncertainty quantification techniques**

Most AI models provide point predictions without confidence estimates, which is a critical requirement for responsible AI in high-stakes medical decision-making.

A prerequisite for efficient clinical translation of AI technology in MASLD is the establishment of uniform data sets, reliable annotation procedures, external verification of results by many institutions, understandable model structures, fairness-aware learning methods, and adherence to the rules and regulations and ethical standards. If all these hurdles can be overcome, the process of collection of data will be very effective, and AI systems will be more credible and universally applicable for clinical purposes.

A further limitation of this review is that a formal risk of bias assessment using standardized tools, such as PROBAST/PROBAST + AI for prediction-model studies and QUADAS-2 for diagnostic accuracy studies, was not performed. Consequently, the methodological quality and potential sources of bias of the included studies were not systematically evaluated. Therefore, the findings should be interpreted with appropriate caution, and future reviews should incorporate standardized risk of bias assessments to strengthen the certainty and reliability of the synthesized evidence.

The characteristics and comparative findings of the included studies have been synthesized across Tables 2–11 according to imaging, clinical, multi-omics, and deployment perspectives. Owing to the broad scoping nature of this review, a single study-level evidence table containing all methodological variables was not developed.

## 7 Conclusion and future work

MASLD has emerged as a pressing global health concern, marked by its deceptive progression and association with metabolic disorders. The complexity of MASLD, from benign steatosis to hepatocellular carcinoma, demands a paradigm shift from conventional diagnostic and prognostic workflows to precision-guided, data-driven solutions. This comprehensive review has underscored the growing influence of AI across the MASLD care continuum spanning clinical, imaging, and multi-omics landscapes.

The integration of AI into imaging has shown promise in enhancing early detection, automating grading, and improving spatial resolution of liver abnormalities. Meanwhile, machine learning applied to clinical and omics data has enabled stratified risk modeling and biomarker discovery with unprecedented depth. However, despite these advancements, the field remains fragmented siloed by modality-specific innovations, region-limited datasets, and a lack of deployment-ready frameworks. The need for interoperability, clinical validation, and fairness-aware models remains critical.

Moving forward, the roadmap for AI in MASLD care must prioritize translational readiness and equitable access. Future research must focus on developing multi-modal architectures capable of jointly analyzing clinical parameters, radiological signals, and molecular phenotypes. AI systems of the future could potentially utilize cascade learning fused with information from multiple modalities. AI systems in the future might use the combination of cascade learning with multi-modal imaging technology, the clinical approach, and multi-omics methodology to better classify the diseases. The information fusion technique established by Delfan et al. serves as a basic principle that can aid in the creation of similar multi-modal AI systems for MASLD.

Approved drugs for MASH have now taken over a more important role for AI in the clinical setting that goes beyond diagnosis and classification. Still, there is a growing demand for AI models to predict eligibility for treatment—especially for patients with significant fibrosis (stages F2–F3)—as well as outcomes of therapy and non-invasive follow-up (63, 73).

There is also an urgent requirement for longitudinal datasets that can power progression-aware models and anticipate transitions across disease stages. Further, FL and privacy-preserving mechanisms need to be mainstreamed, enabling data sharing across healthcare systems without compromising confidentiality. Moreover, explainability will be a non-negotiable component in gaining clinical trust. Developing inherently interpretable models or robust *post-hoc* explainers that resonate with clinician logic will be crucial. Additionally, AI systems must incorporate mechanisms for adaptive learning, wherein post-deployment clinical feedback dynamically refines predictions over time.

Future research should also consider the issue of the cost-effectiveness and budget impact of MASLD care with the aid of AI, in addition to the accuracy of diagnosis. The evidence base illustrating the health economics of AI-assisted MASLD care will be crucial to support its broad implementation, especially in countries with limited healthcare resources (15, 63, 71).

Future MASLD-AI systems must combine technical innovation with practical clinical usability. The success of AI in MASLD will depend on creating human-in-the-loop systems that fit into clinical workflows, meet regulatory standards, and address ethical concerns. By fostering collaborations across clinicians, computer scientists, bioinformaticians, and regulatory bodies, the field can chart a course toward AI systems that are not only smart but safe, scalable, and socially responsible. The next chapter in MASLD research will not be written by AI alone but by its synergistic partnership with the clinical world.
