# Supplementary material for: AI-driven diagnostic and prognostic models for metabolic dysfunction-associated steatotic liver disease: insights from clinical, imaging, and multi-omics studies—a scoping review
Source: Front Med (Lausanne). 2026 Aug 27;13:1875846. doi: 10.3389/fmed.2026.1875846 (PMC13558856; doi:10.3389/fmed.2026.1875846)
Supplement: Supplementary file 1 [file Table1.docx]

**Supplementary Table S1. Database-Specific Search Strategies Used for Literature Retrieval**

| **Database** | **Search Strategy** | **Filters Applied** |
| --- | --- | --- |
| **PubMed** | (("Metabolic Dysfunction-Associated Steatotic Liver Disease"[Title/Abstract] OR MASLD[Title/Abstract] OR "Metabolic Dysfunction Associated Fatty Liver Disease"[Title/Abstract] OR MAFLD[Title/Abstract] OR "Non-Alcoholic Fatty Liver Disease"[MeSH Terms] OR NAFLD[Title/Abstract] OR MASH[Title/Abstract] OR NASH[Title/Abstract]) AND ("Artificial Intelligence"[MeSH Terms] OR "Artificial Intelligence"[Title/Abstract] OR "Machine Learning"[Title/Abstract] OR "Deep Learning"[Title/Abstract] OR Radiomics[Title/Abstract] OR "Medical Imaging"[Title/Abstract] OR "Multi-omics"[Title/Abstract])) | English; Human studies; Publication years: 2020–2026 |
| **Scopus** | TITLE-ABS-KEY(("Metabolic Dysfunction-Associated Steatotic Liver Disease" OR MASLD OR "Metabolic Dysfunction Associated Fatty Liver Disease" OR MAFLD OR "Non-Alcoholic Fatty Liver Disease" OR NAFLD OR MASH OR NASH) AND ("Artificial Intelligence" OR "Machine Learning" OR "Deep Learning" OR Radiomics OR "Medical Imaging" OR "Multi-omics")) | English; Articles & Reviews; Publication years: 2020–2026 |
| **Web of Science** | TS=(("Metabolic Dysfunction-Associated Steatotic Liver Disease" OR MASLD OR "Metabolic Dysfunction Associated Fatty Liver Disease" OR MAFLD OR "Non-Alcoholic Fatty Liver Disease" OR NAFLD OR MASH OR NASH) AND ("Artificial Intelligence" OR "Machine Learning" OR "Deep Learning" OR Radiomics OR "Medical Imaging" OR "Multi-omics")) | English; SCI-Expanded & ESCI; Publication years: 2020–2026 |
| **IEEE Xplore** | (("Metabolic Dysfunction-Associated Steatotic Liver Disease" OR MASLD OR "Metabolic Dysfunction Associated Fatty Liver Disease" OR MAFLD OR "Non-Alcoholic Fatty Liver Disease" OR NAFLD) AND ("Artificial Intelligence" OR "Machine Learning" OR "Deep Learning" OR "Medical Imaging")) | English; Journal Articles & Conference Papers; Publication years: 2020–2026 |

**Note:** Search strategies were developed using controlled vocabulary (MeSH terms where applicable) and free-text keywords. Boolean operators (AND, OR) were adapted according to the syntax requirements of each database. Searches were restricted to English-language publications published between 2020 and 2026.
